# Supplementary material for: Population ageing and mortality during 1990–2017: A global decomposition analysis
Source: PLoS Med. 2020 Jun 8;17(6):e1003138. doi: 10.1371/journal.pmed.1003138 (PMC7279585; doi:10.1371/journal.pmed.1003138)
Supplement: S1 Table — (DOCX) [file pmed.1003138.s002.docx]

**S2 Table. Proportion of people aged 65 years and older in 1990 and 2017**

| **Country and territory** | **Proportion of people aged 65 years and older (%)** | | |
| --- | --- | --- | --- |
|  | **1990** | **2017** | **Difference** |
| Global | 6.1 | 8.8 | 2.7 |
| World Bank High Income | 12.1 | 17.5 | 5.4 |
| World Bank Upper Middle Income | 5.6 | 10.3 | 4.6 |
| World Bank Lower Middle Income | 3.9 | 5.5 | 1.6 |
| World Bank Low Income | 3.2 | 3.1 | -0.1 |
| Afghanistan | 5.5 | 2.2 | -3.4 |
| Albania | 5.0 | 13.7 | 8.6 |
| Algeria | 4.0 | 6.2 | 2.2 |
| American Samoa | 3.3 | 5.3 | 1.9 |
| Andorra | 9.5 | 14.2 | 4.7 |
| Angola | 2.4 | 2.2 | -0.1 |
| Antigua and Barbuda | 8.2 | 9.1 | 0.9 |
| Argentina | 8.7 | 11.0 | 2.2 |
| Armenia | 5.3 | 11.4 | 6.2 |
| Australia | 11.0 | 15.8 | 4.8 |
| Austria | 14.9 | 18.6 | 3.7 |
| Azerbaijan | 4.7 | 5.6 | 0.9 |
| Bahrain | 2.2 | 2.9 | 0.8 |
| Bangladesh | 3.1 | 6.2 | 3.1 |
| Barbados | 11.6 | 14.6 | 3.0 |
| Belarus | 10.6 | 14.7 | 4.1 |
| Belgium | 14.8 | 18.5 | 3.7 |
| Belize | 4.2 | 4.7 | 0.5 |
| Benin | 3.4 | 2.8 | -0.5 |
| Bermuda | 9.1 | 17.1 | 8.0 |
| Bhutan | 3.2 | 4.7 | 1.6 |
| Bolivia | 3.8 | 6.0 | 2.2 |
| Bosnia and Herzegovina | 6.4 | 15.8 | 9.4 |
| Botswana | 3.4 | 4.2 | 0.7 |
| Brazil | 4.6 | 8.8 | 4.2 |
| Brunei | 2.7 | 4.5 | 1.8 |
| Bulgaria | 13.0 | 21.1 | 8.0 |
| Burkina Faso | 3.5 | 2.9 | -0.6 |
| Burundi | 2.7 | 2.2 | -0.5 |
| Cambodia | 3.1 | 5.0 | 2.0 |
| Cameroon | 3.0 | 2.8 | -0.2 |
| Canada | 11.3 | 17.1 | 5.8 |
| Cape Verde | 5.8 | 5.9 | 0.1 |
| Central African Republic | 2.6 | 2.5 | 0.0 |
| Chad | 3.9 | 2.5 | -1.4 |
| Chile | 6.3 | 11.2 | 4.9 |
| China | 5.5 | 11.3 | 5.8 |
| Colombia | 4.1 | 9.0 | 5.0 |
| Comoros | 3.1 | 4.6 | 1.5 |
| Congo | 3.2 | 3.1 | -0.1 |
| Costa Rica | 4.7 | 8.7 | 4.0 |
| Cote d'Ivoire | 2.0 | 2.5 | 0.5 |
| Croatia | 11.2 | 19.4 | 8.2 |
| Cuba | 8.5 | 14.7 | 6.1 |
| Cyprus | 10.1 | 13.8 | 3.7 |
| Czech Republic | 12.5 | 19.3 | 6.8 |
| Democratic Republic of the Congo | 2.8 | 2.6 | -0.2 |
| Denmark | 15.6 | 19.1 | 3.5 |
| Djibouti | 1.6 | 3.0 | 1.4 |
| Dominica | 9.6 | 11.7 | 2.1 |
| Dominican Republic | 4.1 | 7.1 | 3.0 |
| Ecuador | 4.2 | 7.4 | 3.1 |
| Egypt | 3.4 | 3.9 | 0.6 |
| El Salvador | 4.6 | 8.1 | 3.4 |
| Equatorial Guinea | 3.1 | 2.2 | -0.9 |
| Eritrea | 1.7 | 2.1 | 0.4 |
| Estonia | 11.6 | 19.3 | 7.8 |
| Ethiopia | 2.6 | 2.7 | 0.1 |
| Federated States of Micronesia | 3.8 | 3.9 | 0.2 |
| Fiji | 3.1 | 5.6 | 2.5 |
| Finland | 13.5 | 21.4 | 7.9 |
| France | 13.7 | 19.3 | 5.6 |
| Gabon | 4.6 | 4.2 | -0.4 |
| Georgia | 8.9 | 14.6 | 5.6 |
| Germany | 14.9 | 20.9 | 5.9 |
| Ghana | 3.0 | 3.5 | 0.5 |
| Greece | 13.5 | 21.6 | 8.1 |
| Greenland | 3.8 | 8.7 | 4.9 |
| Grenada | 7.2 | 12.8 | 5.7 |
| Guam | 3.9 | 8.6 | 4.7 |
| Guatemala | 3.4 | 5.2 | 1.8 |
| Guinea | 4.5 | 3.4 | -1.1 |
| Guinea-Bissau | 2.9 | 2.4 | -0.5 |
| Guyana | 3.7 | 5.9 | 2.2 |
| Haiti | 3.7 | 3.9 | 0.3 |
| Honduras | 3.4 | 5.0 | 1.6 |
| Hungary | 13.3 | 18.9 | 5.6 |
| Iceland | 10.6 | 14.2 | 3.6 |
| India | 3.6 | 6.2 | 2.6 |
| Indonesia | 3.6 | 5.7 | 2.1 |
| Iran | 3.2 | 6.2 | 2.9 |
| Iraq | 3.4 | 3.8 | 0.4 |
| Ireland | 11.2 | 13.6 | 2.4 |
| Israel | 9.4 | 11.6 | 2.2 |
| Italy | 15.0 | 22.3 | 7.3 |
| Jamaica | 7.1 | 8.7 | 1.6 |
| Japan | 11.9 | 27.5 | 15.6 |
| Jordan | 2.4 | 3.7 | 1.3 |
| Kazakhstan | 5.6 | 7.0 | 1.4 |
| Kenya | 2.6 | 2.9 | 0.3 |
| Kiribati | 3.6 | 3.4 | -0.2 |
| Kuwait | 2.0 | 3.2 | 1.2 |
| Kyrgyzstan | 5.2 | 4.5 | -0.6 |
| Laos | 3.8 | 4.1 | 0.3 |
| Latvia | 11.7 | 20.0 | 8.2 |
| Lebanon | 4.0 | 5.8 | 1.8 |
| Lesotho | 4.3 | 4.7 | 0.4 |
| Liberia | 4.9 | 2.7 | -2.2 |
| Libya | 3.3 | 4.4 | 1.1 |
| Lithuania | 10.6 | 19.3 | 8.8 |
| Luxembourg | 13.0 | 14.3 | 1.2 |
| Macedonia | 7.3 | 13.9 | 6.6 |
| Madagascar | 3.1 | 2.3 | -0.8 |
| Malawi | 3.3 | 3.4 | 0.1 |
| Malaysia | 3.7 | 6.3 | 2.6 |
| Maldives | 2.6 | 4.1 | 1.6 |
| Mali | 3.3 | 2.9 | -0.4 |
| Malta | 10.5 | 20.1 | 9.6 |
| Marshall Islands | 2.7 | 3.3 | 0.6 |
| Mauritania | 4.0 | 3.8 | -0.2 |
| Mauritius | 5.3 | 10.6 | 5.3 |
| Mexico | 3.9 | 7.2 | 3.3 |
| Moldova | 8.2 | 13.2 | 4.9 |
| Mongolia | 3.9 | 3.9 | 0.0 |
| Montenegro | 8.0 | 14.4 | 6.4 |
| Morocco | 4.5 | 6.7 | 2.2 |
| Mozambique | 3.0 | 2.4 | -0.6 |
| Myanmar | 4.2 | 6.2 | 2.0 |
| Namibia | 4.3 | 4.6 | 0.3 |
| Nepal | 3.4 | 5.7 | 2.3 |
| Netherlands | 12.8 | 18.5 | 5.8 |
| New Zealand | 11.0 | 15.8 | 4.9 |
| Nicaragua | 3.1 | 5.5 | 2.4 |
| Niger | 2.4 | 2.3 | -0.1 |
| Nigeria | 3.9 | 2.7 | -1.2 |
| North Korea | 5.6 | 9.9 | 4.3 |
| Northern Mariana Islands | 2.0 | 7.3 | 5.3 |
| Norway | 16.3 | 16.6 | 0.3 |
| Oman | 2.3 | 2.4 | 0.0 |
| Pakistan | 4.1 | 3.5 | -0.6 |
| Palestine | 3.4 | 3.4 | -0.1 |
| Panama | 5.2 | 8.5 | 3.4 |
| Papua New Guinea | 2.6 | 2.5 | -0.1 |
| Paraguay | 4.4 | 6.0 | 1.6 |
| Peru | 4.3 | 7.7 | 3.4 |
| Philippines | 3.3 | 4.9 | 1.5 |
| Poland | 10.0 | 16.7 | 6.6 |
| Portugal | 13.1 | 21.2 | 8.1 |
| Puerto Rico | 9.4 | 18.6 | 9.2 |
| Qatar | 1.0 | 1.1 | 0.1 |
| Romania | 10.2 | 18.3 | 8.0 |
| Russian Federation | 9.8 | 14.0 | 4.2 |
| Rwanda | 2.9 | 3.0 | 0.1 |
| Saint Lucia | 6.0 | 9.8 | 3.7 |
| Saint Vincent and the Grenadines | 6.2 | 10.0 | 3.8 |
| Samoa | 3.7 | 4.9 | 1.1 |
| Sao Tome and Principe | 4.5 | 3.5 | -1.1 |
| Saudi Arabia | 2.6 | 2.4 | -0.2 |
| Senegal | 3.3 | 3.6 | 0.3 |
| Serbia | 9.8 | 17.1 | 7.3 |
| Seychelles | 7.0 | 7.8 | 0.8 |
| Sierra Leone | 4.2 | 3.1 | -1.1 |
| Singapore | 5.5 | 10.1 | 4.5 |
| Slovakia | 10.3 | 15.2 | 4.9 |
| Slovenia | 10.6 | 18.9 | 8.3 |
| Solomon Islands | 2.7 | 3.2 | 0.6 |
| Somalia | 2.0 | 2.5 | 0.5 |
| South Africa | 4.5 | 6.1 | 1.6 |
| South Korea | 4.9 | 13.6 | 8.7 |
| South Sudan | 2.9 | 2.3 | -0.6 |
| Spain | 13.3 | 18.9 | 5.6 |
| Sri Lanka | 4.7 | 9.6 | 4.9 |
| Sudan | 3.7 | 3.2 | -0.5 |
| Suriname | 5.0 | 7.9 | 2.9 |
| Swaziland | 2.5 | 3.7 | 1.1 |
| Sweden | 17.8 | 20.0 | 2.2 |
| Switzerland | 14.3 | 18.1 | 3.8 |
| Syria | 3.0 | 5.1 | 2.1 |
| Taiwan | 6.3 | 13.9 | 7.6 |
| Tajikistan | 3.9 | 3.3 | -0.6 |
| Tanzania | 3.2 | 3.2 | 0.0 |
| Thailand | 4.4 | 11.3 | 6.9 |
| The Bahamas | 4.7 | 7.4 | 2.6 |
| The Gambia | 2.6 | 3.2 | 0.7 |
| Timor-Leste | 2.0 | 5.4 | 3.4 |
| Togo | 2.3 | 3.0 | 0.7 |
| Tonga | 4.4 | 6.3 | 1.9 |
| Trinidad and Tobago | 6.2 | 11.0 | 4.9 |
| Tunisia | 4.8 | 8.5 | 3.7 |
| Turkey | 4.4 | 8.9 | 4.5 |
| Turkmenistan | 3.7 | 4.8 | 1.1 |
| Uganda | 2.8 | 2.3 | -0.5 |
| Ukraine | 11.8 | 15.9 | 4.1 |
| United Arab Emirates | 1.0 | 0.8 | -0.2 |
| United Kingdom | 15.7 | 17.7 | 2.1 |
| United States | 12.4 | 15.4 | 3.0 |
| Uruguay | 11.8 | 14.7 | 2.9 |
| Uzbekistan | 4.0 | 4.0 | 0.0 |
| Vanuatu | 3.1 | 4.1 | 1.0 |
| Venezuela | 3.9 | 7.1 | 3.2 |
| Vietnam | 4.9 | 6.6 | 1.7 |
| Virgin Islands, U.S. | 6.2 | 17.4 | 11.2 |
| Yemen | 2.6 | 2.9 | 0.3 |
| Zambia | 2.5 | 2.4 | -0.1 |
| Zimbabwe | 3.0 | 3.2 | 0.2 |

Note：Proportion of people aged 65 years and older was calculated as “Numbers of people aged 65 years and older / Total numbers of people × 100%”
