# Supplementary material for: Population ageing and mortality during 1990–2017: A global decomposition analysis
Source: PLoS Med. 2020 Jun 8;17(6):e1003138. doi: 10.1371/journal.pmed.1003138 (PMC7279585; doi:10.1371/journal.pmed.1003138)
Supplement: S2 Table — (DOCX) [file pmed.1003138.s003.docx]

**S3 Table. Cause of death with attributed proportion of deaths more than 10%**

| **Male** | | | | | **Female** | | | | |
| --- | --- | --- | --- | --- | --- | --- | --- | --- | --- |
| **Country/territory** | **Cause of death** | **Total deaths in 1990** | **Number of deaths attributed to population ageing** | **Proportion of deaths attributed to population ageing (%)** | **Country/territory** | **Cause of death** | **Total deaths in 1990** | **Number of deaths attribute to population ageing** | **Proportion of deaths attributed to population ageing (%)** |
| Japan | Alzheimer's disease and other types of dementia | 443803 | 46008 | 10.4 | Japan | Alzheimer's disease and other types of dementia | 374876 | 105557 | 28.2 |
| China | Chronic obstructive pulmonary disease | 4407695 | 590229 | 13.4 | Andorra | Alzheimer's disease and other types of dementia | 98 | 22 | 22.5 |
| Botswana | HIV/AIDS | 5492 | 597 | 10.9 | South Korea | Alzheimer's disease and other types of dementia | 99940 | 15702 | 15.7 |
| Albania | Ischemic heart disease | 9671 | 2817 | 29.1 | Spain | Alzheimer's disease and other types of dementia | 155609 | 20784 | 13.4 |
| Virgin Islands, U.S. | Ischemic heart disease | 379 | 106 | 27.9 | Taiwan | Alzheimer's disease and other types of dementia | 40320 | 5284 | 13.1 |
| Bosnia and Herzegovina | Ischemic heart disease | 15417 | 3883 | 25.2 | Italy | Alzheimer's disease and other types of dementia | 259962 | 30441 | 11.7 |
| Malta | Ischemic heart disease | 1396 | 349 | 25.0 | Puerto Rico | Alzheimer's disease and other types of dementia | 10565 | 1209 | 11.4 |
| Guam | Ischemic heart disease | 348 | 85 | 24.5 | Singapore | Alzheimer's disease and other types of dementia | 5985 | 682 | 11.4 |
| Bahrain | Ischemic heart disease | 1153 | 277 | 24.1 | Finland | Alzheimer's disease and other types of dementia | 24922 | 2716 | 10.9 |
| Tunisia | Ischemic heart disease | 25499 | 5893 | 23.1 | Northern Mariana Islands | Alzheimer's disease and other types of dementia | 52 | 6 | 10.7 |
| Armenia | Ischemic heart disease | 13105 | 2926 | 22.3 | Thailand | Alzheimer's disease and other types of dementia | 131393 | 13992 | 10.6 |
| United Arab Emirates | Ischemic heart disease | 3476 | 771 | 22.2 | Greece | Alzheimer's disease and other types of dementia | 38671 | 4034 | 10.4 |
| Mauritius | Ischemic heart disease | 4010 | 845 | 21.1 | Portugal | Alzheimer's disease and other types of dementia | 48302 | 4960 | 10.3 |
| Finland | Ischemic heart disease | 24952 | 5175 | 20.7 | Northern Mariana Islands | Chronic kidney disease | 52 | 6 | 10.8 |
| Bermuda | Ischemic heart disease | 265 | 54 | 20.5 | North Korea | Chronic obstructive pulmonary disease | 64594 | 13192 | 20.4 |
| Algeria | Ischemic heart disease | 67253 | 13602 | 20.2 | China | Chronic obstructive pulmonary disease | 3735430 | 503172 | 13.5 |
| Singapore | Ischemic heart disease | 7690 | 1550 | 20.2 | Fiji | Diabetes mellitus | 1816 | 253 | 14.0 |
| Northern Mariana Islands | Ischemic heart disease | 83 | 16 | 19.4 | Northern Mariana Islands | Diabetes mellitus | 52 | 6 | 11.0 |
| Syria | Ischemic heart disease | 37344 | 7119 | 19.1 | Trinidad and Tobago | Diabetes mellitus | 3726 | 398 | 10.7 |
| Grenada | Ischemic heart disease | 359 | 66 | 18.4 | Lithuania | Ischemic heart disease | 18797 | 7188 | 38.2 |
| Lithuania | Ischemic heart disease | 20552 | 3743 | 18.2 | Virgin Islands, U.S. | Ischemic heart disease | 278 | 85 | 30.6 |
| Costa Rica | Ischemic heart disease | 6577 | 1177 | 17.9 | Bosnia and Herzegovina | Ischemic heart disease | 12528 | 3820 | 30.5 |
| Germany | Ischemic heart disease | 424088 | 75820 | 17.9 | Tunisia | Ischemic heart disease | 18647 | 5576 | 29.9 |
| Iran | Ischemic heart disease | 186033 | 32529 | 17.5 | Bulgaria | Ischemic heart disease | 43175 | 12386 | 28.7 |
| Poland | Ischemic heart disease | 209002 | 36505 | 17.5 | Moldova | Ischemic heart disease | 21569 | 5916 | 27.4 |
| Kuwait | Ischemic heart disease | 3511 | 605 | 17.2 | Armenia | Ischemic heart disease | 11446 | 3123 | 27.3 |
| Australia | Ischemic heart disease | 64476 | 11042 | 17.1 | Guam | Ischemic heart disease | 219 | 59 | 27.0 |
| Cuba | Ischemic heart disease | 40316 | 6837 | 17.0 | Romania | Ischemic heart disease | 115513 | 30909 | 26.8 |
| Japan | Ischemic heart disease | 443803 | 75465 | 17.0 | Malta | Ischemic heart disease | 1294 | 331 | 25.6 |
| Greece | Ischemic heart disease | 49059 | 8269 | 16.9 | Japan | Ischemic heart disease | 374876 | 95128 | 25.4 |
| Canada | Ischemic heart disease | 104239 | 17305 | 16.6 | Estonia | Ischemic heart disease | 9960 | 2382 | 23.9 |
| Estonia | Ischemic heart disease | 9332 | 1550 | 16.6 | Cuba | Ischemic heart disease | 31790 | 7546 | 23.7 |
| South Korea | Ischemic heart disease | 137373 | 22558 | 16.4 | Northern Mariana Islands | Ischemic heart disease | 52 | 12 | 22.5 |
| Croatia | Ischemic heart disease | 27007 | 4412 | 16.3 | Latvia | Ischemic heart disease | 17426 | 3898 | 22.4 |
| Qatar | Ischemic heart disease | 819 | 132 | 16.2 | Andorra | Ischemic heart disease | 98 | 22 | 22.4 |
| Andorra | Ischemic heart disease | 179 | 29 | 16.1 | South Korea | Ischemic heart disease | 99940 | 22281 | 22.3 |
| Montenegro | Ischemic heart disease | 2384 | 382 | 16.0 | Poland | Ischemic heart disease | 179626 | 39227 | 21.8 |
| Czech Republic | Ischemic heart disease | 66627 | 10615 | 15.9 | Mauritius | Ischemic heart disease | 2875 | 620 | 21.6 |
| New Zealand | Ischemic heart disease | 13935 | 2212 | 15.9 | Puerto Rico | Ischemic heart disease | 10565 | 2274 | 21.5 |
| Romania | Ischemic heart disease | 132855 | 21030 | 15.8 | Colombia | Ischemic heart disease | 67710 | 14512 | 21.4 |
| Trinidad and Tobago | Ischemic heart disease | 4557 | 711 | 15.6 | Croatia | Ischemic heart disease | 25262 | 5229 | 20.7 |
| Bulgaria | Ischemic heart disease | 59825 | 9282 | 15.5 | Costa Rica | Ischemic heart disease | 5010 | 1019 | 20.3 |
| Moldova | Ischemic heart disease | 22225 | 3426 | 15.4 | Iran | Ischemic heart disease | 133446 | 26229 | 19.7 |
| Saint Vincent and the Grenadines | Ischemic heart disease | 341 | 52 | 15.4 | Bahrain | Ischemic heart disease | 754 | 147 | 19.5 |
| Georgia | Ischemic heart disease | 26681 | 4068 | 15.2 | Slovakia | Ischemic heart disease | 24731 | 4787 | 19.4 |
| Latvia | Ischemic heart disease | 16677 | 2540 | 15.2 | Bermuda | Ischemic heart disease | 192 | 37 | 19.2 |
| Turkey | Ischemic heart disease | 211001 | 31042 | 14.7 | Algeria | Ischemic heart disease | 54645 | 10425 | 19.1 |
| Venezuela | Ischemic heart disease | 52169 | 7579 | 14.5 | Serbia | Ischemic heart disease | 41669 | 7912 | 19.0 |
| Macedonia | Ischemic heart disease | 8298 | 1193 | 14.4 | Syria | Ischemic heart disease | 25982 | 4942 | 19.0 |
| Russian Federation | Ischemic heart disease | 796183 | 113569 | 14.3 | Albania | Ischemic heart disease | 6948 | 1323 | 19.0 |
| Malaysia | Ischemic heart disease | 47871 | 6754 | 14.1 | Greece | Ischemic heart disease | 38671 | 7308 | 18.9 |
| Fiji | Ischemic heart disease | 2467 | 346 | 14.0 | Georgia | Ischemic heart disease | 26268 | 4939 | 18.8 |
| Ukraine | Ischemic heart disease | 298765 | 41945 | 14.0 | Singapore | Ischemic heart disease | 5985 | 1090 | 18.2 |
| Puerto Rico | Ischemic heart disease | 15268 | 2094 | 13.7 | Taiwan | Ischemic heart disease | 40320 | 7318 | 18.1 |
| Serbia | Ischemic heart disease | 54962 | 7427 | 13.5 | Macedonia | Ischemic heart disease | 6233 | 1076 | 17.3 |
| Slovenia | Ischemic heart disease | 9529 | 1263 | 13.3 | Belarus | Ischemic heart disease | 56384 | 9503 | 16.9 |
| Morocco | Ischemic heart disease | 91728 | 11994 | 13.1 | Slovenia | Ischemic heart disease | 8734 | 1478 | 16.9 |
| Greenland | Ischemic heart disease | 265 | 34 | 12.8 | Panama | Ischemic heart disease | 4304 | 705 | 16.4 |
| Austria | Ischemic heart disease | 38174 | 4802 | 12.6 | Ukraine | Ischemic heart disease | 333123 | 54484 | 16.4 |
| Belarus | Ischemic heart disease | 53867 | 6794 | 12.6 | Maldives | Ischemic heart disease | 592 | 95 | 16.1 |
| Colombia | Ischemic heart disease | 101371 | 12656 | 12.5 | Morocco | Ischemic heart disease | 93151 | 14952 | 16.1 |
| Turkmenistan | Ischemic heart disease | 14276 | 1783 | 12.5 | Venezuela | Ischemic heart disease | 38921 | 6235 | 16.0 |
| Taiwan | Ischemic heart disease | 64112 | 7950 | 12.4 | Sri Lanka | Ischemic heart disease | 36518 | 5819 | 15.9 |
| Iceland | Ischemic heart disease | 917 | 113 | 12.3 | Trinidad and Tobago | Ischemic heart disease | 3726 | 591 | 15.9 |
| Saint Lucia | Ischemic heart disease | 475 | 57 | 12.1 | Finland | Ischemic heart disease | 24922 | 3923 | 15.7 |
| Slovakia | Ischemic heart disease | 30454 | 3689 | 12.1 | Czech Republic | Ischemic heart disease | 62348 | 9668 | 15.5 |
| Panama | Ischemic heart disease | 5810 | 687 | 11.8 | Turkey | Ischemic heart disease | 159296 | 24385 | 15.3 |
| Portugal | Ischemic heart disease | 52931 | 6224 | 11.8 | Canada | Ischemic heart disease | 88337 | 13169 | 14.9 |
| Italy | Ischemic heart disease | 281279 | 32800 | 11.7 | Portugal | Ischemic heart disease | 48302 | 7153 | 14.8 |
| Jordan | Ischemic heart disease | 8498 | 990 | 11.7 | Spain | Ischemic heart disease | 155609 | 22708 | 14.6 |
| Lebanon | Ischemic heart disease | 12533 | 1445 | 11.5 | Brazil | Ischemic heart disease | 394698 | 57095 | 14.5 |
| Netherlands | Ischemic heart disease | 66656 | 7672 | 11.5 | Hungary | Ischemic heart disease | 68306 | 9920 | 14.5 |
| Cyprus | Ischemic heart disease | 2953 | 337 | 11.4 | Australia | Ischemic heart disease | 55056 | 7782 | 14.1 |
| Thailand | Ischemic heart disease | 184625 | 20772 | 11.3 | Dominican Republic | Ischemic heart disease | 17099 | 2414 | 14.1 |
| United States | Ischemic heart disease | 1111057 | 122744 | 11.0 | Italy | Ischemic heart disease | 259962 | 36655 | 14.1 |
| Brunei | Ischemic heart disease | 636 | 68 | 10.7 | Israel | Ischemic heart disease | 13967 | 1956 | 14.0 |
| Spain | Ischemic heart disease | 176000 | 18805 | 10.7 | Russian Federation | Ischemic heart disease | 841216 | 114579 | 13.6 |
| Ireland | Ischemic heart disease | 16676 | 1770 | 10.6 | Thailand | Ischemic heart disease | 131393 | 17913 | 13.6 |
| Libya | Ischemic heart disease | 10524 | 1108 | 10.5 | Chile | Ischemic heart disease | 34847 | 4670 | 13.4 |
| Dominican Republic | Ischemic heart disease | 22154 | 2308 | 10.4 | Lebanon | Ischemic heart disease | 9730 | 1268 | 13.0 |
| The Bahamas | Ischemic heart disease | 790 | 81 | 10.3 | Montenegro | Ischemic heart disease | 2006 | 259 | 12.9 |
| Chile | Ischemic heart disease | 43901 | 4464 | 10.2 | Brunei | Ischemic heart disease | 436 | 53 | 12.2 |
| China | Ischemic heart disease | 4407695 | 449249 | 10.2 | Malaysia | Ischemic heart disease | 36896 | 4467 | 12.1 |
| Sri Lanka | Ischemic heart disease | 68828 | 7003 | 10.2 | El Salvador | Ischemic heart disease | 13432 | 1601 | 11.9 |
| Japan | Lower respiratory infections | 443803 | 54528 | 12.3 | Cyprus | Ischemic heart disease | 2476 | 278 | 11.2 |
| Albania | Stroke | 9671 | 2157 | 22.3 | Libya | Ischemic heart disease | 8077 | 900 | 11.1 |
| South Korea | Stroke | 137373 | 29726 | 21.6 | Fiji | Ischemic heart disease | 1816 | 201 | 11.0 |
| China | Stroke | 4407695 | 724013 | 16.4 | Saint Vincent and the Grenadines | Ischemic heart disease | 361 | 39 | 10.9 |
| Japan | Stroke | 443803 | 71846 | 16.2 | Grenada | Ischemic heart disease | 394 | 42 | 10.8 |
| Northern Mariana Islands | Stroke | 83 | 13 | 15.9 | Mexico | Ischemic heart disease | 184926 | 19533 | 10.6 |
| Portugal | Stroke | 52931 | 8340 | 15.8 | Iceland | Ischemic heart disease | 839 | 88 | 10.5 |
| Macedonia | Stroke | 8298 | 1233 | 14.9 | China | Ischemic heart disease | 3735430 | 393461 | 10.5 |
| Montenegro | Stroke | 2384 | 344 | 14.4 | New Zealand | Ischemic heart disease | 12784 | 1324 | 10.4 |
| Bosnia and Herzegovina | Stroke | 15417 | 2097 | 13.6 | Kuwait | Ischemic heart disease | 1772 | 182 | 10.3 |
| Greece | Stroke | 49059 | 5951 | 12.1 | Germany | Ischemic heart disease | 493533 | 50471 | 10.2 |
| Taiwan | Stroke | 64112 | 7755 | 12.1 | Turkmenistan | Ischemic heart disease | 12325 | 1236 | 10.0 |
| North Korea | Stroke | 60224 | 7193 | 11.9 | Japan | Lower respiratory infections | 374876 | 43819 | 11.7 |
| Grenada | Stroke | 359 | 41 | 11.5 | Singapore | Lower respiratory infections | 5985 | 655 | 10.9 |
| Thailand | Stroke | 184625 | 20325 | 11.0 | Andorra | Lower respiratory infections | 98 | 10 | 10.6 |
| Mauritius | Stroke | 4010 | 439 | 10.9 | South Korea | Stroke | 99940 | 29135 | 29.2 |
| Serbia | Stroke | 54962 | 5979 | 10.9 | Japan | Stroke | 374876 | 96678 | 25.8 |
|  |  |  |  |  | Macedonia | Stroke | 6233 | 1521 | 24.4 |
|  |  |  |  |  | Portugal | Stroke | 48302 | 10828 | 22.4 |
|  |  |  |  |  | Bosnia and Herzegovina | Stroke | 12528 | 2804 | 22.4 |
|  |  |  |  |  | Montenegro | Stroke | 2006 | 430 | 21.4 |
|  |  |  |  |  | Taiwan | Stroke | 40320 | 8465 | 21.0 |
|  |  |  |  |  | Albania | Stroke | 6948 | 1359 | 19.6 |
|  |  |  |  |  | Northern Mariana Islands | Stroke | 52 | 10 | 19.6 |
|  |  |  |  |  | Greece | Stroke | 38671 | 7509 | 19.4 |
|  |  |  |  |  | Serbia | Stroke | 41669 | 7668 | 18.4 |
|  |  |  |  |  | Bulgaria | Stroke | 43175 | 7832 | 18.1 |
|  |  |  |  |  | Romania | Stroke | 115513 | 20903 | 18.1 |
|  |  |  |  |  | China | Stroke | 3735430 | 603196 | 16.1 |
|  |  |  |  |  | Andorra | Stroke | 98 | 15 | 15.5 |
|  |  |  |  |  | North Korea | Stroke | 64594 | 9620 | 14.9 |
|  |  |  |  |  | Thailand | Stroke | 131393 | 17952 | 13.7 |
|  |  |  |  |  | Latvia | Stroke | 17426 | 2373 | 13.6 |
|  |  |  |  |  | Tunisia | Stroke | 18647 | 2476 | 13.3 |
|  |  |  |  |  | Mauritius | Stroke | 2875 | 353 | 12.3 |
|  |  |  |  |  | Croatia | Stroke | 25262 | 2930 | 11.6 |
|  |  |  |  |  | Singapore | Stroke | 5985 | 679 | 11.3 |
|  |  |  |  |  | Georgia | Stroke | 26268 | 2932 | 11.2 |
|  |  |  |  |  | Slovenia | Stroke | 8734 | 980 | 11.2 |
|  |  |  |  |  | Spain | Stroke | 155609 | 17376 | 11.2 |
|  |  |  |  |  | Guam | Stroke | 219 | 24 | 11.0 |
|  |  |  |  |  | Sri Lanka | Stroke | 36518 | 4016 | 11.0 |
|  |  |  |  |  | Malta | Stroke | 1294 | 139 | 10.7 |
|  |  |  |  |  | Saint Lucia | Stroke | 419 | 44 | 10.6 |
|  |  |  |  |  | Brazil | Stroke | 394698 | 41781 | 10.6 |
|  |  |  |  |  | Malaysia | Stroke | 36896 | 3915 | 10.6 |
|  |  |  |  |  | Panama | Stroke | 4304 | 456 | 10.6 |
|  |  |  |  |  | Lithuania | Stroke | 18797 | 1967 | 10.5 |
|  |  |  |  |  | Italy | Stroke | 259962 | 27211 | 10.5 |
|  |  |  |  |  | Brunei | Stroke | 436 | 45 | 10.3 |
|  |  |  |  |  | Colombia | Stroke | 67710 | 6935 | 10.2 |
|  |  |  |  |  | Virgin Islands, U.S. | Stroke | 278 | 28 | 10.2 |
|  |  |  |  |  | Armenia | Stroke | 11446 | 1144 | 10.0 |

Note: Proportion of deaths attributed to population ageing was calculated as “Deaths attributed to population ageing / All causes male (female) deaths in 1990 × 100%” for each country and cause of death.
