# Supplementary material for: Population ageing and mortality during 1990–2017: A global decomposition analysis
Source: PLoS Med. 2020 Jun 8;17(6):e1003138. doi: 10.1371/journal.pmed.1003138 (PMC7279585; doi:10.1371/journal.pmed.1003138)
Supplement: S3 Table — (DOCX) [file pmed.1003138.s004.docx]

**S3 Table.** Comparative contributions of mortality reduction versus population ageing to change in number of deaths between 1990 and 2017.

| **Male** | | | | |  | **Female** | | | | |
| --- | --- | --- | --- | --- | --- | --- | --- | --- | --- | --- |
| **Country/ territory** | **Deaths attributed to population growth** | **Deaths attributed to population ageing** | **Deaths attributed to mortality change** | ***R*** |  | **Country/ territory** | **Deaths attributed to population growth** | **Deaths attributed to population ageing** | **Deaths attributed to mortality change** | ***R*** |
| Global | 9616574 | 6951894 | -11077519 | -1.6 |  | Global | 8390559 | 5607946 | -10022038 | -1.8 |
| World Bank High Income | 930991 | 2284283 | -2327085 | -1.0 |  | World Bank High Income | 764585 | 2091487 | -1836484 | -0.9 |
| World Bank Upper Middle Income | 2017008 | 4470943 | -3766182 | -0.8 |  | World Bank Upper Middle Income | 1749963 | 3714763 | -3888669 | -1.0 |
| World Bank Lower Middle Income | 5118561 | 1193560 | -4507443 | -3.8 |  | World Bank Lower Middle Income | 4566894 | 1185764 | -4384223 | -3.7 |
| Albania | -2449 | 9546 | -3386 | -0.4 |  | Albania | -1195 | 4537 | -1915 | -0.4 |
| Algeria | 36966 | 28700 | -43347 | -1.5 |  | Algeria | 30783 | 24095 | -31188 | -1.3 |
| American Samoa | 19 | 52 | -29 | -0.6 |  | American Samoa | 17 | 40 | 8 | 0.2 |
| Andorra | 84 | 132 | -91 | -0.7 |  | Andorra | 72 | 127 | -41 | -0.3 |
| Antigua and Barbuda | 102 | 69 | -101 | -1.5 |  | Antigua and Barbuda | 86 | 28 | -4 | -0.1 |
| Argentina | 45001 | 32460 | -45871 | -1.4 |  | Argentina | 39878 | 41542 | -34083 | -0.8 |
| Armenia | -1940 | 7341 | -4018 | -0.5 |  | Armenia | -1368 | 7452 | -3724 | -0.5 |
| Australia | 27326 | 40720 | -43386 | -1.1 |  | Australia | 24641 | 30425 | -28385 | -0.9 |
| Austria | 5913 | 17324 | -22302 | -1.3 |  | Austria | 4552 | 14323 | -21449 | -1.5 |
| Azerbaijan | 12938 | 3976 | -1413 | -0.4 |  | Azerbaijan | 8725 | 330 | -3949 | -12.0 |
| Bahrain | 2009 | 603 | -1678 | -2.8 |  | Bahrain | 1039 | 423 | -975 | -2.3 |
| Bangladesh | 193297 | 110603 | -441153 | -4.0 |  | Bangladesh | 191772 | 47410 | -382515 | -8.1 |
| Barbados | 194 | 422 | -387 | -0.9 |  | Barbados | 199 | 281 | -233 | -0.8 |
| Belarus | -5942 | 16220 | -4519 | -0.3 |  | Belarus | -5346 | 19682 | -9406 | -0.5 |
| Belgium | 7149 | 21814 | -27833 | -1.3 |  | Belgium | 6664 | 18706 | -22135 | -1.2 |
| Belize | 573 | 108 | -62 | -0.6 |  | Belize | 435 | 33 | -183 | -5.5 |
| Bermuda | 27 | 179 | -162 | -0.9 |  | Bermuda | 26 | 140 | -141 | -1.0 |
| Bhutan | 1400 | 317 | -1784 | -5.6 |  | Bhutan | 1154 | 295 | -2146 | -7.3 |
| Bolivia | 20915 | 5980 | -24760 | -4.1 |  | Bolivia | 18711 | 7121 | -24788 | -3.5 |
| Bosnia and Herzegovina | -5414 | 12940 | -4094 | -0.3 |  | Bosnia and Herzegovina | -4248 | 12066 | -2185 | -0.2 |
| Botswana | 3825 | 1273 | -3266 | -2.6 |  | Botswana | 2681 | 1239 | -707 | -0.6 |
| Brazil | 224796 | 257177 | -290897 | -1.1 |  | Brazil | 179876 | 262895 | -243784 | -0.9 |
| Brunei | 424 | 304 | -306 | -1.0 |  | Brunei | 334 | 328 | -281 | -0.9 |
| Bulgaria | -14966 | 23986 | -12546 | -0.5 |  | Bulgaria | -10757 | 31209 | -12306 | -0.4 |
| Cambodia | 26737 | 6229 | -37717 | -6.1 |  | Cambodia | 21910 | 12198 | -40197 | -3.3 |
| Canada | 34903 | 65213 | -62905 | -1.0 |  | Canada | 31892 | 53222 | -35497 | -0.7 |
| Cape Verde | 716 | 129 | -484 | -3.8 |  | Cape Verde | 475 | 128 | -368 | -2.9 |
| Chile | 15734 | 25629 | -26544 | -1.0 |  | Chile | 13563 | 26743 | -21556 | -0.8 |
| China | 824805 | 3164067 | -2344116 | -0.7 |  | China | 745309 | 2555523 | -2636608 | -1.0 |
| Colombia | 51064 | 46632 | -71594 | -1.5 |  | Colombia | 40434 | 57750 | -59149 | -1.0 |
| Comoros | 1118 | 129 | -1570 | -12.2 |  | Comoros | 997 | 369 | -1500 | -4.1 |
| Congo | 13196 | 1150 | -11777 | -10.2 |  | Congo | 11832 | 365 | -6971 | -19.1 |
| Costa Rica | 3859 | 4768 | -1571 | -0.3 |  | Costa Rica | 3379 | 4397 | -2868 | -0.7 |
| Croatia | -3753 | 14811 | -12720 | -0.9 |  | Cote d'Ivoire | 49257 | 796 | -32429 | -40.7 |
| Cuba | 1872 | 23026 | -10132 | -0.4 |  | Croatia | -3555 | 15530 | -11371 | -0.7 |
| Cyprus | 1783 | 1273 | -1473 | -1.2 |  | Cuba | 2340 | 24849 | -12617 | -0.5 |
| Czech Republic | 2791 | 29966 | -43655 | -1.5 |  | Cyprus | 1486 | 1227 | -1987 | -1.6 |
| Denmark | 3513 | 8300 | -15026 | -1.8 |  | Czech Republic | 1051 | 26791 | -35419 | -1.3 |
| Djibouti | 2230 | 569 | -1377 | -2.4 |  | Denmark | 2869 | 5204 | -10745 | -2.1 |
| Dominica | -15 | 106 | -14 | -0.1 |  | Djibouti | 1816 | 346 | -1193 | -3.4 |
| Dominican Republic | 11685 | 6821 | -794 | -0.1 |  | Dominica | -30 | 73 | -16 | -0.2 |
| Ecuador | 19143 | 11273 | -11484 | -1.0 |  | Dominican Republic | 7487 | 7552 | -4952 | -0.7 |
| Egypt | 159779 | 20151 | -104787 | -5.2 |  | Ecuador | 15412 | 10907 | -9140 | -0.8 |
| El Salvador | 2291 | 6175 | -4654 | -0.8 |  | Egypt | 106688 | 10681 | -119106 | -11.2 |
| Eritrea | 29567 | 332 | -53513 | -161.2 |  | El Salvador | 2903 | 6245 | -4437 | -0.7 |
| Estonia | -1458 | 3845 | -4639 | -1.2 |  | Estonia | -1705 | 5297 | -5336 | -1.0 |
| Federated States of Micronesia | -2 | 72 | -79 | -1.1 |  | Federated States of Micronesia | 3 | 72 | -87 | -1.2 |
| Fiji | 551 | 1079 | -87 | -0.1 |  | Fiji | 430 | 1075 | -77 | -0.1 |
| Finland | 3148 | 15662 | -16064 | -1.0 |  | Finland | 2234 | 13282 | -13064 | -1.0 |
| France | 36847 | 142382 | -162016 | -1.1 |  | France | 37490 | 132435 | -134637 | -1.0 |
| Gabon | 3299 | 219 | -2785 | -12.7 |  | Georgia | -10703 | 11693 | -2117 | -0.2 |
| Georgia | -10394 | 10047 | -912 | -0.1 |  | Germany | 10822 | 172365 | -193089 | -1.1 |
| Germany | 27254 | 249090 | -236753 | -1.0 |  | Ghana | 58728 | 4366 | -39569 | -9.1 |
| Greece | -557 | 32858 | -18579 | -0.6 |  | Greece | 304 | 31469 | -12969 | -0.4 |
| Greenland | -4 | 165 | -145 | -0.9 |  | Greenland | 7 | 88 | -99 | -1.1 |
| Grenada | 135 | 299 | -259 | -0.9 |  | Grenada | 100 | 212 | -99 | -0.5 |
| Guam | 91 | 285 | 4 | 0.0 |  | Guam | 86 | 216 | 4 | 0.0 |
| Guatemala | 33569 | 4749 | -25035 | -5.3 |  | Guatemala | 27750 | 6286 | -26881 | -4.3 |
| Guyana | -156 | 892 | -726 | -0.8 |  | Guyana | -111 | 797 | -442 | -0.6 |
| Honduras | 13032 | 3176 | -8905 | -2.8 |  | Haiti | 27455 | 607 | -24967 | -41.1 |
| Hungary | -5721 | 22072 | -33244 | -1.5 |  | Honduras | 11716 | 2942 | -4863 | -1.7 |
| Iceland | 314 | 365 | -351 | -1.0 |  | Hungary | -4269 | 30155 | -32165 | -1.1 |
| India | 2197900 | 939535 | -2387983 | -2.5 |  | Iceland | 261 | 351 | -493 | -1.4 |
| Indonesia | 272822 | 181633 | -268558 | -1.5 |  | India | 2070051 | 1014246 | -2307136 | -2.3 |
| Iran | 70586 | 78200 | -116081 | -1.5 |  | Indonesia | 219303 | 224307 | -333549 | -1.5 |
| Iraq | 75480 | 6564 | -53967 | -8.2 |  | Iran | 52673 | 61599 | -86450 | -1.4 |
| Ireland | 5032 | 6056 | -12005 | -2.0 |  | Iraq | 57484 | 714 | -50826 | -71.2 |
| Israel | 11363 | 5239 | -9181 | -1.8 |  | Ireland | 4721 | 4483 | -8783 | -2.0 |
| Italy | 19830 | 161509 | -164014 | -1.0 |  | Israel | 10993 | 8491 | -10284 | -1.2 |
| Jamaica | 1441 | 2244 | 544 | 0.2 |  | Italy | 19223 | 184266 | -139925 | -0.8 |
| Japan | 6489 | 520990 | -267453 | -0.5 |  | Jamaica | 1183 | 2193 | -559 | -0.3 |
| Jordan | 13486 | 2225 | -7909 | -3.6 |  | Japan | 16032 | 575505 | -298509 | -0.5 |
| Kazakhstan | 4150 | 15287 | -14830 | -1.0 |  | Jordan | 10405 | 1526 | -8126 | -5.3 |
| Kiribati | 221 | 15 | -92 | -6.1 |  | Kazakhstan | 3881 | 6165 | -9546 | -1.5 |
| Kuwait | 3880 | 1518 | -3311 | -2.2 |  | Kenya | 78237 | 3953 | -40723 | -10.3 |
| Kyrgyzstan | 7223 | 725 | -8649 | -11.9 |  | Kiribati | 189 | 28 | -124 | -4.4 |
| Latvia | -5318 | 6543 | -4659 | -0.7 |  | Kuwait | 2095 | 545 | -2277 | -4.2 |
| Lebanon | 11481 | 3174 | -9977 | -3.1 |  | Laos | 12582 | 381 | -20334 | -53.4 |
| Lesotho | 809 | 187 | 2940 | 15.7 |  | Latvia | -5388 | 9349 | -6576 | -0.7 |
| Libya | 7079 | 2716 | -321 | -0.1 |  | Lebanon | 9314 | 3574 | -6252 | -1.7 |
| Lithuania | -6333 | 8923 | -2995 | -0.3 |  | Lesotho | 736 | 701 | 2653 | 3.8 |
| Luxembourg | 942 | 571 | -1406 | -2.5 |  | Libya | 5558 | 2274 | -1451 | -0.6 |
| Macedonia | 801 | 4549 | -2092 | -0.5 |  | Lithuania | -5196 | 13552 | -6402 | -0.5 |
| Malaysia | 40176 | 27050 | -19052 | -0.7 |  | Luxembourg | 849 | 432 | -807 | -1.9 |
| Maldives | 702 | 106 | -816 | -7.7 |  | Macedonia | 552 | 4434 | -3665 | -0.8 |
| Malta | 308 | 1093 | -768 | -0.7 |  | Malaysia | 27341 | 20599 | -15746 | -0.8 |
| Marshall Islands | 43 | 58 | -36 | -0.6 |  | Maldives | 348 | 324 | -819 | -2.5 |
| Mauritius | 680 | 3035 | -1998 | -0.7 |  | Malta | 252 | 1122 | -704 | -0.6 |
| Mexico | 123438 | 115217 | -84497 | -0.7 |  | Marshall Islands | 30 | 31 | -8 | -0.3 |
| Moldova | -4104 | 8237 | -4298 | -0.5 |  | Mauritius | 601 | 2362 | -1232 | -0.5 |
| Mongolia | 4690 | 1441 | -3867 | -2.7 |  | Mexico | 97071 | 106139 | -77996 | -0.7 |
| Montenegro | -17 | 1413 | -390 | -0.3 |  | Moldova | -3887 | 11455 | -9392 | -0.8 |
| Morocco | 35467 | 20180 | -33978 | -1.7 |  | Mongolia | 3831 | 1055 | -5560 | -5.3 |
| Myanmar | 61459 | 41974 | -142898 | -3.4 |  | Montenegro | 18 | 1164 | -77 | -0.1 |
| Namibia | 4129 | 384 | -2049 | -5.3 |  | Morocco | 34013 | 30940 | -50789 | -1.6 |
| Nepal | 42064 | 13941 | -65667 | -4.7 |  | Myanmar | 59810 | 50826 | -146349 | -2.9 |
| Netherlands | 9912 | 35327 | -38175 | -1.1 |  | Namibia | 3351 | 1086 | -2339 | -2.2 |
| New Zealand | 3954 | 7755 | -8882 | -1.1 |  | Nepal | 46079 | 11803 | -80566 | -6.8 |
| Nicaragua | 5859 | 2444 | -6917 | -2.8 |  | Netherlands | 9100 | 25189 | -17566 | -0.7 |
| North Korea | 24335 | 28160 | 754 | 0.0 |  | New Zealand | 4063 | 5539 | -6526 | -1.2 |
| Northern Mariana Islands | -4 | 92 | -7 | -0.1 |  | Nicaragua | 4834 | 2794 | -5315 | -1.9 |
| Norway | 5351 | 3940 | -12784 | -3.2 |  | North Korea | 17133 | 42691 | -2828 | -0.1 |
| Oman | 6601 | 154 | -4487 | -29.1 |  | Northern Mariana Islands | 2 | 76 | -28 | -0.4 |
| Panama | 3935 | 2896 | -2066 | -0.7 |  | Norway | 4299 | 3252 | -7955 | -2.4 |
| Papua New Guinea | 31022 | 1992 | -9456 | -4.7 |  | Oman | 2525 | 282 | -2515 | -8.9 |
| Paraguay | 7465 | 3065 | -1773 | -0.6 |  | Panama | 3143 | 2967 | -2012 | -0.7 |
| Peru | 33514 | 23658 | -55743 | -2.4 |  | Papua New Guinea | 21575 | 903 | -6640 | -7.4 |
| Philippines | 146249 | 43638 | -37047 | -0.8 |  | Paraguay | 5799 | 2951 | -2751 | -0.9 |
| Poland | -5335 | 102480 | -102457 | -1.0 |  | Peru | 26921 | 21494 | -45685 | -2.1 |
| Portugal | 2283 | 36200 | -34520 | -1.0 |  | Philippines | 103360 | 55046 | -30862 | -0.6 |
| Puerto Rico | 2 | 9094 | -5934 | -0.7 |  | Poland | -1893 | 107048 | -94120 | -0.9 |
| Qatar | 3758 | 282 | -1904 | -6.8 |  | Portugal | 3755 | 39951 | -35148 | -0.9 |
| Romania | -27177 | 63474 | -32831 | -0.5 |  | Puerto Rico | 392 | 9921 | -4790 | -0.5 |
| Russian Federation | -36758 | 309651 | -150565 | -0.5 |  | Qatar | 995 | 82 | -661 | -8.1 |
| Saint Lucia | 170 | 329 | -253 | -0.8 |  | Romania | -22129 | 86420 | -55022 | -0.6 |
| Saint Vincent and the Grenadines | 27 | 249 | -38 | -0.2 |  | Russian Federation | -24230 | 291607 | -192381 | -0.7 |
| Samoa | 89 | 118 | -107 | -0.9 |  | Rwanda | 26428 | 1244 | -48599 | -39.1 |
| Serbia | -2838 | 25461 | -20432 | -0.8 |  | Saint Lucia | 121 | 253 | -183 | -0.7 |
| Seychelles | 141 | 88 | -108 | -1.2 |  | Saint Vincent and the Grenadines | 4 | 173 | -112 | -0.6 |
| Singapore | 4938 | 6500 | -7792 | -1.2 |  | Samoa | 88 | 124 | -4 | 0.0 |
| Slovakia | 737 | 10188 | -14696 | -1.4 |  | Senegal | 26206 | 1062 | -27088 | -25.5 |
| Slovenia | 602 | 6308 | -6423 | -1 |  | Serbia | -3557 | 26400 | 2020 | 0.1 |
| Solomon Islands | 1186 | 150 | -535 | -3.6 |  | Seychelles | 74 | 62 | -34 | -0.5 |
| South Africa | 81782 | 28945 | -9688 | -0.3 |  | Singapore | 5129 | 5008 | -7618 | -1.5 |
| South Korea | 30308 | 158858 | -163407 | -1.0 |  | Slovakia | 727 | 11414 | -10773 | -0.9 |
| Spain | 30988 | 100528 | -96874 | -1.0 |  | Slovenia | 184 | 6701 | -5730 | -0.9 |
| Sri Lanka | 13688 | 28452 | -37811 | -1.3 |  | Solomon Islands | 908 | 293 | -415 | -1.4 |
| Suriname | 704 | 514 | -267 | -0.5 |  | South Africa | 64935 | 33560 | -4231 | -0.1 |
| Swaziland | 1582 | 355 | 474 | 1.3 |  | South Korea | 22676 | 129021 | -115155 | -0.9 |
| Sweden | 8306 | 11512 | -23621 | -2.1 |  | Spain | 29565 | 116648 | -95945 | -0.8 |
| Switzerland | 7104 | 12378 | -20599 | -1.7 |  | Sri Lanka | 12288 | 27797 | -23763 | -0.9 |
| Syria | 19199 | 10440 | 8615 | 0.8 |  | Suriname | 615 | 487 | -412 | -0.8 |
| Taiwan | 9186 | 61131 | -28242 | -0.5 |  | Swaziland | 1024 | 493 | 176 | 0.4 |
| Thailand | 46993 | 144906 | -103815 | -0.7 |  | Sweden | 6665 | 8853 | -14196 | -1.6 |
| The Bahamas | 391 | 426 | -232 | -0.5 |  | Switzerland | 6573 | 9727 | -13190 | -1.4 |
| Timor-Leste | 1993 | 755 | -2641 | -3.5 |  | Syria | 11656 | 7807 | -6661 | -0.9 |
| Tonga | 19 | 83 | -14 | -0.2 |  | Taiwan | 11027 | 54429 | -32327 | -0.6 |
| Trinidad and Tobago | 815 | 2713 | -1607 | -0.6 |  | Tanzania | 124318 | 1365 | -122855 | -90.0 |
| Tunisia | 9091 | 13400 | -9782 | -0.7 |  | Thailand | 39204 | 126441 | -102519 | -0.8 |
| Turkey | 74372 | 85869 | -124868 | -1.5 |  | The Bahamas | 330 | 296 | -97 | -0.3 |
| Turkmenistan | 5355 | 3466 | -4440 | -1.3 |  | The Gambia | 4072 | 272 | -2107 | -7.7 |
| Ukraine | -56255 | 92167 | 19992 | 0.2 |  | Timor-Leste | 1819 | 636 | -2959 | -4.7 |
| United Arab Emirates | 17257 | 2366 | -1437 | -0.6 |  | Togo | 15166 | 841 | -11198 | -13.3 |
| United Kingdom | 52175 | 101232 | -170421 | -1.7 |  | Tonga | 20 | 111 | -60 | -0.5 |
| United States | 332355 | 415315 | -397297 | -1.0 |  | Trinidad and Tobago | 627 | 2415 | -1837 | -0.8 |
| Uruguay | 1219 | 3671 | -4535 | -1.2 |  | Tunisia | 7640 | 13567 | -11799 | -0.9 |
| Uzbekistan | 39562 | 1616 | 5798 | 3.6 |  | Turkey | 57589 | 74049 | -135014 | -1.8 |
| Vanuatu | 629 | 152 | -139 | -0.9 |  | Turkmenistan | 3486 | 2488 | -3963 | -1.6 |
| Venezuela | 37262 | 24907 | -10651 | -0.4 |  | Ukraine | -54018 | 107281 | -51177 | -0.5 |
| Vietnam | 106283 | 88789 | -84104 | -0.9 |  | United Arab Emirates | 4028 | 363 | -1263 | -3.5 |
| Virgin Islands, U.S. | -16 | 386 | 6 | 0.0 |  | United Kingdom | 41472 | 52408 | -114575 | -2.2 |
| Zimbabwe | 18324 | 898 | -2823 | -3.1 |  | United States | 290235 | 269341 | -192357 | -0.7 |
|  |  |  |  |  |  | Uruguay | 1500 | 5450 | -3856 | -0.7 |
|  |  |  |  |  |  | Vanuatu | 416 | 136 | -86 | -0.6 |
|  |  |  |  |  |  | Venezuela | 26437 | 23447 | -19907 | -0.8 |
|  |  |  |  |  |  | Vietnam | 74345 | 84835 | -97089 | -1.1 |
|  |  |  |  |  |  | Virgin Islands, U.S. | 2 | 307 | -85 | -0.3 |
|  |  |  |  |  |  | Zambia | 45029 | 402 | -48000 | -119.4.0 |
|  |  |  |  |  |  | Zimbabwe | 16017 | 3102 | -61 | 0.0 |

Notes: Deaths attributed to population growth, population ageing and mortality change was calculated by decomposition method; *R* was calculated as “Deaths attributed to mortality change / Deaths attributed to population ageing”.
