# Supplementary material for: Population ageing and mortality during 1990–2017: A global decomposition analysis
Source: PLoS Med. 2020 Jun 8;17(6):e1003138. doi: 10.1371/journal.pmed.1003138 (PMC7279585; doi:10.1371/journal.pmed.1003138)
Supplement: S1 Text — (DOCX) [file pmed.1003138.s005.docx]

**The decomposition method**

All decomposition methods for absolute numbers attribute differences or changes in total deaths to the changes of various components, or factors, such as population size, age structure and mortality rates. The new decomposition method we adopted in this study was reported to be robust to the choice of decomposition order of the three factors and the choice of reference group, compared to the two most commonly used decomposition methods [1].

Using the difference in total deaths in 1990 and in 2017 for the world as an example, we demonstrate the calculation of deaths attributed to the three factors. Age was divided using 5-year increments, from under-5 years old to 95 years and older. Let *d_ij_*, *n_ij_*, *m_ij_* and *s_ij_* denote the number of deaths, population size, age-specific mortality rate, and proportion of population for the *i*^th^ age group of the *j*^th^ year, respectively, (*i* = 1, 2, …, 20; *j* = 1, 2). Let *D*_1_ and *D*_2_, *N*_1_ and *N*_2_, *M*_1_ and *M*_2_ represent the total number of deaths, population size and crude mortality rate for years 1990 and 2017, respecitvely (**Table 1**).

**Table 1. Meaning of mathematical symbols in the decomposition formula**

| Age group | 1990 (*j*=1) | | | |  | 2017 (*j*=2) | | | |
| --- | --- | --- | --- | --- | --- | --- | --- | --- | --- |
|  | Death | Population | Mortality | Age structure |  | Death | Population | Mortality | Age structure |
| 0-4 | *d*_11_ | *n*_11_ | *m*_11_ | *s*_11_ |  | *d*_12_ | *n*_12_ | *m*_12_ | *s*_12_ |
| 5-9 | *d*_21_ | *n*_21_ | *m*_21_ | *s*_21_ |  | *d*_22_ | *n*_22_ | *m*_22_ | *s*_22_ |
| 10-14 | *d*_31_ | *n*_31_ | *m*_31_ | *s*_31_ |  | *d*_32_ | *n*_32_ | *m*_32_ | *s*_32_ |
| ⁞ | ⁞ | ⁞ | ⁞ | ⁞ |  | ⁞ | ⁞ | ⁞ | ⁞ |
| 90-94 | *d*_191_ | *n*_191_ | *m*_191_ | *s*_191_ |  | *d*_192_ | *n*_192_ | *m*_192_ | *s*_192_ |
| ≥95 | *d*_201_ | *n*_201_ | *m*_201_ | *s*_201_ |  | *d*_202_ | *n*_202_ | *m*_202_ | *s*_202_ |
| Total | *D*_1_ | *N*_1_ | *M*_1_ | *S*_1_=1 |  | *D*_2_ | *N*_2_ | *M*_2_ | *S*_2_=1 |

$D_{1}=\sum_{i=1}^{20} d_{i1}$

$D_{2}=\sum_{i=1}^{20} d_{i2}$

$N_{1}=\sum_{i=1}^{20} n_{i1}$

$N_{2}=\sum_{i=1}^{20} n_{i2}$

$M_{1}={D_{1}}/{N_{1}}$

$M_{2}={D_{2}}/{N_{2}}$

$m_{ij}={d_{ij}}/{n_{ij}}$

$s_{ij}={n_{ij}}/{N_{j}}$

Using *M_p_*, *M_a_* and *M_m_* to represent the main effects of the changes in population size, in age structure and in mortality rates, and *I_pa_*, *I_pm_*, *I_am_* and *I_pam_* to represent their two-way and three-way interactions, respectively. These terms are calculated as follows when using year 1990 as the reference:

$M_{p}=\sum_{i=1}^{20} {{\left( N_{2}-N_{1} \right)s}_{i1}m}_{i1}$

$M_{a}=\sum_{i=1}^{20} N_{1}\left( s_{i2}-s_{i1} \right)m_{i1}$

$M_{m}=\sum_{i=1}^{20} {N_{1}s}_{i1}\left( m_{i2}-m_{i1} \right)$

$I_{pa}=\sum_{i=1}^{20} \left( N_{2}-N_{1} \right)\left( s_{i2}-s_{i1} \right)m_{i1}$

$I_{pm}=\sum_{i=1}^{20} \left( N_{2}-N_{1} \right)s_{i1}\left( m_{i2}-m_{i1} \right)$

$I_{am}=\sum_{i=1}^{20} N_{1}\left( s_{i2}-s_{i1} \right)\left( m_{i2}-m_{i1} \right)$

$I_{pam}=\sum_{i=1}^{20} \left( N_{2}-N_{1} \right)\left( s_{i2}-s_{i1} \right)\left( m_{i2}-m_{i1} \right)$

Using year 2017 as the reference, the formulas are calculated as follows:

$M_{p}^{'}=\sum_{i=1}^{p} {{\left( N_{1}-N_{2} \right)s}_{i2}m}_{i2}$

$M_{a}^{'}=\sum_{i=1}^{p} N_{2}\left( s_{i1}-s_{i2} \right)m_{i2}$

$M_{m}^{'}=\sum_{i=1}^{p} {N_{2}s}_{i2}\left( m_{i1}-m_{i2} \right)$

$I_{pa}^{'}=\sum_{i=1}^{p} \left( N_{1}-N_{2} \right)\left( s_{i1}-s_{i2} \right)m_{i2}$

$I_{pm}^{'}=\sum_{i=1}^{p} \left( N_{1}-N_{2} \right)s_{i2}\left( m_{i1}-m_{i2} \right)$

$I_{am}^{'}=\sum_{i=1}^{p} N_{2}\left( s_{i1}-s_{i2} \right)\left( m_{i1}-m_{i2} \right)$

$I_{pam}^{'}=\sum_{i=1}^{p} \left( N_{1}-N_{2} \right)\left( s_{i1}-s_{i2} \right)\left( m_{i1}-m_{i2} \right)$

The contribution of each factor includes its main effect and partial interactions with other factors.

(1) Suppose *a*%, *b*% and *c*% of the two-way interaction between population size and age structure, population size and mortality change, and age structure and mortality change are allocated to the first factor, respectively. Accordingly, (100-*a*)%, (100-*b*)% and (100-*c*)% of the three two-way interactions are allocated to the second factor.

And (2) suppose *d*_1_%, *d*_2_% and (100- *d*_1_-*d*_2_)% of the three-way interaction are allocated to population size, age structure, and mortality change, respectively.

Using $A$ ($A^{'}$), $M$ ($M^{'}$) and $P$ ($P^{'}$) to represent the number of deaths attributed to age structure, mortality change and population size defined by the method when using year 1990 (year 2017) as reference, the contributions of the three factors can be calculated as follows:

$$P=M_{p}+a\%I_{pa}+b\%I_{pm}+d_{1}\%I_{pam}$$

$${A=M}_{a}+\left( 100-a \right)\%I_{pa}+c\%I_{am}+d_{2}\%I_{pam}$$

$$M=M_{m}+\left( 100-b \right)\%I_{pm}+\left( 100-c \right)\%I_{am}+\left( 100-d_{1}-d_{2} \right)\%I_{pam}$$

$${P^{'}=M}_{p}^{'}+a\%I_{pa}^{'}+b\%I_{pm}^{'}+d_{1}\%I_{pam}^{'}$$

$${A^{'}=M}_{a}^{'}+\left( 100-a \right)\%I_{pa}^{'}+c\%I_{am}^{'}+d_{2}\%I_{pam}^{'}$$

$${M^{'}=M}_{m}^{'}+\left( 100-b \right)\%I_{pm}^{'}+(100-c)\%I_{sm}^{'}+\left( 100-d_{1}-d_{2} \right)\%I_{pam}^{'}$$

The decomposition results should remain unchanged in absolute value when the reference population changes, so we have a group of three equations:

$\left\{ \begin{aligned} P\equiv-P^{'} \\ A\equiv-A^{'} \\ M\equiv-M^{'} \end{aligned} \right.$

Through formula derivation, we have three simplified equations:

$\left\{ \begin{aligned} \sum_{i=1}^{p} \left( N_{2}-N_{1} \right)\left[ \left( s_{i1}m_{i1}-s_{i2}m_{i2} \right)\left( 100-a-b \right)\%+\left( s_{i2}m_{i1}-s_{i1}m_{i2} \right)\left( a-b \right)\% \right]\equiv0 \\ \sum_{i=1}^{p} \left( s_{i2}-s_{i1} \right)\left[ \left( {N_{2}m_{i1}-N}_{1}m_{i2} \right)\left( 100-a-c \right)\%+\left( N_{1}m_{i1}-N_{2}m_{i2} \right)\left( a-c \right)\% \right]\equiv0 \\ \sum_{i=1}^{p} \left( m_{i2}-m_{i1} \right)\left[ \left( {{N_{2}s_{i2}-N}_{1}s}_{i1} \right)\left( 100-b-c \right)\%+\left( N_{1}s_{i2}-N_{2}s_{i1} \right)\left( b-c \right)\% \right]\equiv0 \end{aligned} \right.$

These three equations cannot be true all the time unless *a*, *b*, and *c* all equal 50.

The three equations have no requirements for *d*_1_ and *d*_2_. Given there is no theoretical guidance to allocate the three-way interaction of three factors, we divide it equally, *d*_1_=*d*_2_=⅓×100.

The contributions of the three factors can be calculated as follows:

$A{=M}_{a}+½I_{am}+½I_{pa}+⅓I_{pam}$

$P{=M}_{p}+½I_{pm}+½I_{pa}+⅓I_{pam}$

$M{=M}_{m}+½I_{pm}+½I_{am}+⅓I_{pam}$

Thus, *A* represents the effect of changes in age structure. Because the proportion of older age groups has been reported to increase recently for most countries [2], the effect of age structure represents the effect of population ageing [3-5].

**An example**

The number of population and deaths from China in 1990 and 2017 are provided in Table 2.

**Table 2. The number of population and death from China in 1990 and 2017**

| Age group | China in 1990 | | |  | China in 2017 | | |
| --- | --- | --- | --- | --- | --- | --- | --- |
|  | Deaths | Mortality | Population |  | Deaths | Mortality | Population |
| < 5 | 1317 | 10.2 | 128597 |  | 165 | 2.1 | 80431 |
| 5-9 | 138 | 1.3 | 104984 |  | 28 | 0.4 | 72115 |
| 10-14 | 77 | 0.7 | 102431 |  | 25 | 0.3 | 73460 |
| 15-19 | 99 | 0.8 | 127020 |  | 26 | 0.3 | 76954 |
| 20-24 | 162 | 1.2 | 132196 |  | 36 | 0.4 | 91633 |
| 25-29 | 178 | 1.6 | 110179 |  | 85 | 0.7 | 122996 |
| 30-34 | 184 | 2.1 | 88362 |  | 104 | 0.9 | 119344 |
| 35-39 | 210 | 2.3 | 91534 |  | 98 | 1.0 | 97355 |
| 40-44 | 241 | 3.6 | 67241 |  | 175 | 1.6 | 110812 |
| 45-49 | 261 | 5.0 | 51705 |  | 356 | 2.9 | 124852 |
| 50-54 | 337 | 7.0 | 47798 |  | 538 | 4.5 | 119486 |
| 55-59 | 437 | 10.1 | 43457 |  | 447 | 5.6 | 80552 |
| 60-64 | 621 | 17.5 | 35446 |  | 946 | 11.4 | 82786 |
| 65-69 | 768 | 28.1 | 27368 |  | 1231 | 20.1 | 61210 |
| 70-74 | 943 | 50.0 | 18862 |  | 1206 | 29.6 | 40756 |
| 75-79 | 875 | 76.6 | 11422 |  | 1425 | 50.5 | 28243 |
| 80-84 | 748 | 127.9 | 5847 |  | 1640 | 90.0 | 18234 |
| 85-89 | 406 | 197.6 | 2054 |  | 1199 | 143.6 | 8344 |
| 90-94 | 120 | 286.2 | 421 |  | 571 | 231.0 | 2473 |
| ≥95 | 23 | 402.3 | 57 |  | 150 | 338.4 | 445 |
| Total | 8143 | 6.8 | 1196980 |  | 10452 | 7.4 | 1412480 |

Note: Mortality means age-specific mortality per 1000 persons. Deaths and population are presented in 1000.

The number of deaths attribute to population ageing can be calculated as follows:

$M_{a}=\sum_{i=1}^{20} N_{1}\left( s_{i2}-s_{i1} \right)m_{i1}=1196980\times\left[ \left( \frac{80431}{1412480}-\frac{128597}{1196980} \right)\times10.2+\cdots+\left( \frac{445}{1412480}-\frac{57}{1196980} \right)\times402.3 \right]=6037335.921$

$I_{pa}=\sum_{i=1}^{20} \left( N_{2}-N_{1} \right)\left( s_{i2}-s_{i1} \right)m_{i1}=\left( 1412480-1196980 \right)\times\left[ \left( \frac{80431}{1412480}-\frac{128597}{1196980} \right)\times10.2+\cdots+\left( \frac{445}{1412480}-\frac{57}{1196980} \right)\times402.3 \right]=1086940.376$

$I_{am}=\sum_{i=1}^{20} N_{1}\left( s_{i2}-s_{i1} \right)\left( m_{i2}-m_{i1} \right)=1196980\times\left[ \left( \frac{80431}{1412480}-\frac{128597}{1196980} \right)\times\left( 2.1-10.2 \right)+\cdots+\left( \frac{445}{1412480}-\frac{57}{1196980} \right)\times\left( 338.4-402.3 \right) \right]=-1592755.509$

$I_{pam}=\sum_{i=1}^{20} \left( N_{2}-N_{1} \right)\left( s_{i2}-s_{i1} \right)\left( m_{i2}-m_{i1} \right)=\left( 1412480-1196980 \right)\times\left[ \left( \frac{80431}{1412480}-\frac{128597}{1196980} \right)\times\left( 2.1-10.2 \right)+\cdots+\left( \frac{445}{1412480}-\frac{57}{1196980} \right)\times\left( 338.4-402.3 \right) \right]=-286754.008$

$A{=M}_{a}+½I_{am}+½I_{pa}+⅓I_{pam}=6037335.921-½\times1592755.509+½\times1086940.376-⅓\times286754.008=5688843.685$

The number of deaths attributed to population growth and change of mortality rate can be calculated similarly using the formulas above.

**References**

1. Cheng XJ, Tan LH, Gao YY, Yang Y, Schwebel DC, Hu GQ. A new method to attribute differences in total deaths between groups to population size, age structure and age-specific mortality rate. PLoS One. 2019;14(5):e0216613.

2. Lutz W, Sanderson W, Scherbov S. The coming acceleration of global population ageing. Nature. 2008;451(7179):716-719.

3. Chang AY, Skirbekk VF, Tyrovolas S, Kassebaum NJ, Dieleman JL. Measuring population ageing: an analysis of the Global Burden of Disease Study 2017. Lancet Public Health. 2019;4(3):e159-e167.

4. Global Burden of Disease Cancer C, Fitzmaurice C, Akinyemiju TF, Al Lami FH, Alam T, Alizadeh-Navaei R, et al. Global, regional, and national cancer incidence, mortality, years of life lost, years lived with disability, and disability-adjusted life-years for 29 cancer groups, 1990 to 2016: a systematic analysis for the Global Burden of Disease Study. JAMA Oncol. 2018;4(11):1553-1568.

5. GBD 2015 Mortality and Causes of Death Collaborators. Global, regional, and national life expectancy, all-cause mortality, and cause-specific mortality for 249 causes of death, 1980-2015: a systematic analysis for the Global Burden of Disease Study 2015. Lancet. 2016;388(10053):1459-1544.
