# Supplementary material for: Population ageing and mortality during 1990–2017: A global decomposition analysis
Source: PLoS Med. 2020 Jun 8;17(6):e1003138. doi: 10.1371/journal.pmed.1003138 (PMC7279585; doi:10.1371/journal.pmed.1003138)
Supplement: Attachment — Submitted filename: Response to reviewers.docx [file pmed.1003138.s006.docx]

February 11, 2020

Dear Dr. Gaynor-Brook,

Many thanks for the excellent comments from you and the four reviewers. We greatly appreciate your time. Based on the requests and comments, we have worked to improve the manuscript. In particular, we improved the abstract and discussion as suggested. We believe the manuscript is improved. Please find a point-by-point response to the editor’ requests and the reviewers’ comments below.

We hope the revised manuscript addresses the concerns of the editor and reviewers.

Yours sincerely,

Correspondence to:

Prof. Guoqing Hu

Department of Epidemiology and Health Statistics

Xiangya School of Public Health, Central South University.

110 Xiangya Road, Changsha, 410078, China

Email: [huguoqing009@gmail.com](mailto:huguoqing009@gmail.com)

On behalf of all authors

**Part A: Reply to comments from the editor**

Associate Editor Comments to the Author

General comments:

**Comment 1**. The flow of the main text is, at times, difficult to follow. It would be appreciated if your manuscript could be proofread thoroughly by someone with full professional proficiency in English prior to resubmission.

**Response: Thanks. As suggested,** **our co-author who has published widely in the field and is a native English speaker reviewed the work again to improve the English writing.** **Please see changes to the revised manuscript.**

**Comment 2**. Please remove indents from the beginning of paragraphs.

**Response: Thanks. As suggested, we have removed indents from the beginning of paragraphs.**

**Comment 3**. Please revise your title according to PLOS Medicine's style, placing the study design in the subtitle (ie, after a colon). We suggest "Population ageing and mortality during 1990-2017: a global cross-sectional analysis" or similar.

**Response: Thanks. As suggested, we have revised the title of the article. Please see changes (lines 1-2) to the revised manuscript.**

**Comment 4**. Abstract Background: Please expand upon the context of why the study is important. The final sentence should clearly state the study question.

**Response: Thanks for your good suggestion. We have improved the Background to address the two concerns.** **Please see changes (lines 21-25) to the revised manuscript.**

**Comment 5**. Please combine the Methods and Findings components of your Abstract under ‘Methods and Findings’

**Response: Thanks. As suggested, we have combined the Methods and Findings into a single part. Please see changes (lines 26-51) to the revised manuscript.**

**Comment 6**. Please include the study design, number of individuals included in the GBD 2017 dataset, and brief demographic details e.g. age, sex, distribution of data according to income categories, etc.

**Response: Thanks. We have added brief information about the study population in the abstract; more details are presented in the results.** **Please see changes (lines 34-36, and lines 226-237) to the revised manuscript.**

**Comment 7**. Please expand upon the results relating to variation due to economic development levels and geographic regions

**Response: Thanks for the good suggestion. We have expanded the details for variations due to economic development levels and geographic regions.** **Please see changes (lines 37-41) to the revised manuscript.**

**Comment 8**. Line 38 - Please report your results for increases and decreases in deaths in separate sentences.

**Response: Thanks. As suggested, we have separated reported increases and decreases in deaths into different sentences.** **Please see changes (lines 44-46) to the revised manuscript.**

**Comment 9**. Please include the important dependent variables that are adjusted for in the analyses.

**Response: Thanks. The decomposition method is designed to attribute the change in total deaths to three factors (population growth, population ageing and change of mortality rate). It does not involve adjustment of other independent variables. In fact, the change of mortality rate reflects the combined effects of all relevant factors (excluding population growth and population ageing). As suggested by the reviewers, we have improved the description of the decomposition method to clarify this issue. Please see changes (lines 170-183) to the revised manuscript.**

**Comment 10**. In the last sentence of the Abstract Methods and Findings section, please describe the main limitation(s) of the study's methodology.

**Response: Thanks for good suggestion. We have added the main limitation of the study in the final sentence.** **Please see changes (lines 49-51) to the revised manuscript.**

**Comment 11**. Please begin your Abstract Conclusions with “"In this study, we observed ..." or similar. Please address the study implications substantiated by the results, emphasizing what is new without overstating your conclusions.

**Response:** **Thanks. As suggested, we have improved the conclusions and study implications of abstract.** **Please see changes (lines 52-58) to the revised manuscript.**

**Comment 12**. At this stage, we ask that you include a short, non-technical Author Summary of your research to make findings accessible to a wide audience that includes both scientists and non-scientists. The Author Summary should immediately follow the Abstract in your revised manuscript. This text is subject to editorial change and should use non-identical language distinct from the scientific abstract. Please see our author guidelines for more information: https://journals.plos.org/plosmedicine/s/revising-your-manuscript#loc-author-summary

**Response: Thanks. We have included a short author summary in the revised manuscript.** **Please see changes (lines 62-98) to the revised manuscript.**

Introduction

**Comment 13**. Please address past research and explain the need for and potential importance of your study. Indicate whether your study is novel and how you determined that. If there has been a systematic review of the evidence related to your study (or you have conducted one), please refer to and reference that review and indicate whether it supports the need for your study.

**Response: Thanks. We have improved the summary of past research and emphasize the need for this research. Based on our careful literature search, there is no such a systematic review that can be cited currently.** **Please see changes (lines 113-130) to the revised manuscript.**

**Comment 14**. Line 65 - please expand upon the importance of an increase in global disability adjusted life years (DALYs)

**Response: Thanks for good suggestion. We have expanded this point as suggested.** **Please see changes (lines 109-112) to the revised manuscript.**

**Comment 15**. Line 88 - please revise ‘currently lacking’ and ‘novel’ to avoid assertions of primacy

**Response: Thanks. We have made the changes as suggested.** **Please see changes (lines 131-135) to the revised manuscript.**

Methods

**Comment 16**. Please report your data according to GATHER and enclose a completed GATHER checklist as a supplementary document. See http://gather-statement.org/ In the checklist please include sufficient text excerpted from the manuscript to explain how you accomplished all applicable items. When completing the checklist, please use section titles and paragraph numbers, rather than page numbers.

**Response: Thanks. We have checked the manuscript according to the GATHER checklist and include a completed GATHER checklist as S1 Table. Please see changes in the supplementary table.**

**Comment 17**. Did your study have a prospective protocol or analysis plan? Please state this (either way) early in the Methods section. If a prospective analysis plan was used in designing the study, please include the relevant prospectively written document with your revised manuscript as a Supporting Information file to be published alongside your study, and cite it in the Methods section. If no such document exists, please make sure that the Methods section transparently describes when analyses were planned, and when/why any data-driven changes to analyses took place.

**Response: Thanks. There was no prospective protocol or analysis plan for this study. As suggested, we have included the details of the data analyses, including the timing for them, in the Method section. Our analyses were not data-driven.** **Please see changes (lines 217-221) to the revised manuscript.**

Results

**Comment 18**. Please include as Table 1 a summary of the number of individuals included in the GBD 2017 dataset, and baseline demographic data e.g. age, sex, distribution of data according to income categories, etc.

**Response: Thanks for good suggestion. We have included the information of GBD 2017 dataset as Table 1 as suggested.** **Please see changes (lines 243-244) to the revised manuscript.**

**Comment 19**. Line 203 - please revise to ‘bronchial’

**Response:** **Thanks. As suggested,** **we have changed it.**

**Comment 20**. Line 219 - please expand upon how changes in male deaths “can be explained by increases in the percentage of younger population”

**Response: Thanks for good suggestion. The statement is based on S2 Table, which shows increases in the proportion of people aged 65 years and older in most low income countries. It is well known that all-cause mortality is higher in older people than in younger people. Based on these two facts, it is reasonable to draw the conclusion above. We have improved the relevant sentences to clarify our point. Please see changes (lines 272-275) to the revised manuscript.**

**Comment 21**. Line 242 - please revise sentence beginning ‘Fourteen (23) countries’ to better distinguish between results presented for males and females

**Response:** **Many thanks for pointing out the poorly-written sentence. We have revised the sentence as suggested.** **Please see changes (lines 323-325) to the revised manuscript.**

**Comment 22**. Lines 253, 254 - please revise to ‘other types of dementia’

**Response:** **Thanks. We have changed** **it as suggested.**

**Comment 23**. Lines 255 - 261: please clarify which sex these results apply to

**Response: Thanks for good suggestion. We have improved the writing to specify it.** **Please see changes (lines 334-342) to the revised manuscript.**

**Comment 24**. Figure 3 - Please explain why range of ‘negative proportions’ (shown in blue) does not extend to zero, and why range of first quintile of the positive proportions begins at 0.7% / 0.8% (and not 0.1)

**Response: Thanks. You are right. The positive proportion could start from 0.1, and the negative proportions could end at zero. We originally marked 0.7/0.8 as the lower bound for the second category and -0.9%/-0.8% as the upper bound for the first category in in this figure because 0.7 and 0.8 are the lowest positive observed proportions in the second category, and** **-0.9%/-0.8% are the largest negative proportions in the first category. We agree with your suggestion and have changed the labels in Figure 3 to make the ranges continuous.** **Please see changes to the revised Figure 3.**

**Comment 25**. Figure 4 - please revise title to “Ratios between total deaths attributed to mortality change and deaths attributed to population ageing…”

**Response: Thanks. As suggested, we have changed the title of Figure 4.** **Please see changes (lines 373-374) to the revised manuscript.**

**Comment 26**. Please present numerators and denominators for percentages in the Tables.

**Response: Many thanks for the good suggestion. In Table 2, we now present numerators with the percentages and specify the common denominator in a note under the table. We present both numerators and denominators together with the percentages in S3 Table. Please see changes (lines 296-299) to the revised manuscript.**

**Comment 27**. Table 1 - please revise to ‘bronchial’

**Response:** **Thanks. We have changed it as suggested.**

**Comment 28**. Table 2 - please revise to ‘other types of dementia’ ‘bronchial’ and ‘pulmonary’

**Response: Thanks. As suggested, we have changed them.**

**Comment 28**. Table S2 - please revise to ‘other types of dementia’

**Response:** **Thanks. As suggested, we have changed it in Table S2.**

Discussion

**Comment 29**. Please remove subheadings from within your Discussion.

**Response: Thanks. As suggested, we have removed subheadings in Discussion.**

**Comment 30**. Please begin your Discussion with "In this study” or similar

**Response: Thanks for good suggestion. We have changed it as requested. Please see changes (line 384) to the revised manuscript.**

**Comment 31**. Please present and organize the Discussion as follows: a short, clear summary of the article's findings; what the study adds to existing research and where and why the results may differ from previous research; strengths and limitations of the study; implications and next steps for research, clinical practice, and/or public policy; one-paragraph conclusion.

**Response: Thanks for good suggestion. We have improved the organization of discussion as suggested. Please see changes to the revised manuscript.**

**Comment 32**. Line 315 - please revise to ‘have caused’

**Response: Thanks. As suggested, we have changed “cause” to “have caused”.**

**Comment 33**. Line 352 - please revise ‘high-risk age groups differ across diseases’

**Response: Thanks. As suggested, we have improved the organization of discussion as suggested, and we have dropped this sentence.** **Please see changes to the revised manuscript.**

**Part B: Reply to the comments from Reviewer #1**

Reviewer#1: Comments to the Author

I mostly confine my remarks to statistical aspects of this paper.

**Comment 1**. However, as someone who is new to this idea of disagregating these effects, I was a little unclear as to the purpose of the whole exercise. I guess other people already know this, but, since the audience for PLoS will surely include other people like me, it would be good to give a bit more about this in the introduction. I can certainly see why we would want to know, e.g., projections of disease rates and counts in various countries. But I'm not so clear as to the need for knowing what proportion of the number of deaths is due to aging vs. other causes.

**Response: Thanks for the good suggestion. As requested, we have included more materials in the introduction on the background and value of decomposition of total death burden to make it easier for the general audience to read.** **Please see changes (lines 109-112, and lines 117-130) to the revised manuscript.**

**Comment 2**. Now, to statistics. These were generally fine. I would like to have a bit more detail in the appendix about the derivation of the formulas. I spent a good bit of time figuring out what was being done and I think it is correct, but the authors could help by spelling out how the various formulas were derived and why they are the way they are.

**Response: Thanks. As requested, we have included more information about the decomposition method in the appendix. Please see changes to the revised S1 Text.**

**Comment 3**. Line 45-47: I'm not sure how this follows from the results. I don't disagree with the conclusion, but couldn't we have concluded this a priori? What possible results would make this conclusion incorrect?

**Response: Thanks. We have improved the description of the conclusions to make them match the results more clearly. Please see changes (lines 52-58) to the revised manuscript.**

**Comment 4**. Line 101-102 Some detail of what was done would be good.

**Response: Thanks. Given the space limit and because the details on how raw data are handled are included in GBD publications (eg, GBD 2017 Causes of Death Collaborators, Lancet, 2018), we choose not repeat the complete details in this manuscript. We do include a short paragraph to specify the information sources.** **Please see changes (lines 147-152) to the revised manuscript.**

*GBD 2017 Causes of Death Collaborators. Global, regional, and national age-sex-specific mortality for 282 causes of death in 195 countries and territories, 1980-2017: a systematic analysis for the Global Burden of Disease Study 2017. Lancet. 2018;392(10159):1736–1788.*

**Comment 5**. Line 120-124: It won't really affect anything, but I think the subscripts and formula labels could be better chosen, just to make it easier to follow. There are three factors: Aging, population growth and mortality change. Why not use A, P and M for these, both instead of A, B and C and instead of S, P and M (in the subscripts)?

**Response: Many thanks. As suggested, we have changed the subscripts and formula labels.** **Please see changes (lines 170-186) to the revised manuscript.**

**Comment 6**. Fig 1 Stacked bar charts (which is really what this is) aren't a great method. See the work of William S. Cleveland. I would use a line graph with year on the x axis, deaths on the y axis and three or four lines (one for each cause and maybe one for total)

**Response: Thanks for the good suggestion. We have redone Figure 1 with a line graph.** **Please see changes to Figure 1.**

**Comment 7**. Fig 2: First, the y axis can't be proportion of deaths - proportions have to add to 1.00 (or, percentages to 100). I'm not sure what is being graphed here. Is it number of deaths? Second, I'd put the labels for the lines next to the lines on the right axis for easier reading.

**Response: Thanks for the good suggestion. We are sorry for our unthoughtful choice of labels. The y axis is referring to the relative contribution of population ageing, which was estimated as “the number of attributed deaths divided by the total number of deaths in 1990 × 100%”. We have changed the expression of y-axis and the labels of Figure 2.** **Please see changes to Figure 2.**

**Part C: Reply to the comments from Reviewer #2**

Reviewer#2: Comments to the Author

**Comment 1**. This work tries to provide a better understanding of the drivers causing (old age) mortality (line 22-23). Conditional on the choice of their models, there is no reason to dispute the data presented. Questions arise however on the framing of work, the underlying assumptions, and the interpretation of the data.

**Response: Many thanks. We answer the three questions point by point below.**

Framing

**Comment 2**. This work is positioned in a reasoning that 'ageing is a global public health challenge' (line 21-22). The direct connect between population ageing and public health challenge however, is a postulation, not a fact. The idea that aging is a (negative) 'challenge' is adhered to by many, but the question is whether it is correct, and or helpful. The key is that a decrease in the force of mortality is a good thing: it is getting better for individuals. It indicates societal progress and achievements, and allows for longer investments into work, society, others. Rephrased otherwise, population ageing can equally be framed as something positive, e.g. the 'silver economy'. A negative preconception could well be considered a form of ageism.

**Response:** **Many thanks for your thoughtful comments. We fully agree with you that old persons contribute to society in many positive ways and that older adults should enjoy and appreciate life. At the same time, it is indisputable that older individuals have more physical health challenges than younger individuals, and this does create a public health burden on societies that must be addressed as society ages. Given the reviewer’s concern, which we agree with, we have carefully reviewed the manuscript to avoid any implication of ageism and to focus only on our academic goals of quantifying the impact of population ageing on the number of deaths and identifying possible target countries and diseases for future intervention, without implying societal aging is a negative occurrence.** **Please see changes (lines 21-25, and lines 226-237) to the revised manuscript.**

Assumptions

**Comment 3**. Not separating the average age of the population and age specific force of mortality can make up every outcome one can think of. It is the explanation why different methods come up with inconsistent results (line 112-117). It is for these reasons that the authors take an absolute stance, 'attributing differences or changes in total deaths to the changes of various components, or factors, such as population size, age structure and mortality rates' (line 117 onwards). The methodology followed is correct when the absolute number of deaths is the primary endpoint to be considered, e.g. Figure 1. However, it is questionable whether the decomposition method provides 'the robust model' that is claimed to unravel the drivers of mortality. It can easily be deduced from the figure that the change in total deaths is the net result of population size, force of mortality and age structure, phenomena that are interrelated, but very different between countries depending on what happened over successive generations, calendar time and lifespan. The model allows for including these various interactions, but the consequence is that outcomes of the model are only weighted averages, and different for the whole world, regions, and each country separately. When very strict, this underlying heterogeneity between countries and regions would prohibit statistical pooling…

**Response: Many thanks for raising great comments. The robustness of the method means that the method is insensitive to changes of decomposition order of the three factors (population size, age structure, and age-specific mortality) or the choice of a reference group, compared to previously published methods (Cheng et al., 2019, PLoS One). Different studies may use the same traditional method but with different decomposition orders or reference groups and thus render the results not comparable to each other. We have revised the text in the methods section to clarify this point about “robustness”. In addition, we agree with the reviewer that** **t****he decomposition method has its limitations and does not consider underlying heterogeneity in factors not included in the decomposition, e.g., changes in age structure may vary between countries and regions. Nevertheless, we think the global decomposition results are still valuable for policy-makers. In addition, we have performed subpopulation-specific analyses by country, sex and income level, partially accounting for such heterogeneity. We have discussed this limitation and what we have done to overcome this limitation to some extent, and suggest future research for methodological improvement.** **Please see changes (lines 445-451) to the revised manuscript.**

**Comment 4**. A similar reasoning can be set up when classifying a nation as an "ageing country", even when it is according to the United Nations standard (proportion of people ≥ 65 years old exceeds 7%). Given the huge differences in age specific mortality, morbidity, functionality (e.g. pension age varies between 55 to none) 7% can be appreciated as low, preferred, or high. Moreover, the use of 'ageing countries' is ambivalent as there may be different underlying processes going on. In extreme, one could argue that some nations that have successfully dealt with early mortality at young age are now finally ageing, which is a positive thing. This is not a semantic discussion, e.g. a careful exploration and presentation of these separate phenomena unmasks the double burden of disease, and explains why these nations can only prosper economically when their populations are ageing.

**Response: Many thanks. We agree and have dropped the sentences involving “ageing countries” to avoid unintended interpretation.**

Interpretation

**Comment 5**. It is very difficult to infer 'global trend in disease-related deaths attributed to population ageing from 1990 to 2017' (line 399). The reason is that the underlying demographic dynamics are so different:

-	First, in some countries it could be that: As the number of new-borns decreases, population ageing accelerates as a global public health challenge. The interpretation being that it is the decrease in fertility that is the underlying problem and should be addressed, which reasoning, correct or wrong, is now being followed in several countries of the world;

-	Second, in other countries 'We face as a global public health challenge, as the number of deaths increases because the of baby boom.' This could well be a true phenomenon but the interpretation is not negative. See that some argue for active family planning to elicit a baby boom, thus wanted. Moreover, at the same time force of mortality could well be going down, thus it is not a public health problem but a late effect of an earlier societal behaviour;

-	Third, for specific countries;' Life expectancy increases, population ageing accelerates, indicating that a global public health challenge is successfully dealt with.' For example, life expectancy in Russia dropped because there was a massive alcohol problem. Now this is partially dealt with, the population is ageing again, which is positive.

**Response: Thanks for the insightful comments. We agree with the reviewer that it is difficult to interpret trends in disease-related deaths as attributed to population ageing from 1990 to 2017. The trends have many explanations, just as the three possibilities raised by the reviewer, and the interpretation may differ across countries. We also agree that it is difficult and inappropriate to label the trends as positive or negative. However, it is also a fact that the increase in the proportion of older adults in many nations means we must consider how to prepare health care systems to respond to the relatively higher mortality and morbidity risks among older populations. To avoid misleading readers and to acknowledge the possibilities raised by the reviewer, we have carefully rephrased the explanations of our findings and the limitations of our analyses.** **Please see changes (lines 420-425, and lines 442-444) to the revised manuscript.**

**Comment 6**. In conclusion, disaggregation of the impact of ageing on death is a research priority and can best be dealt with when population size, age structure and mortality rates are dealt with in all countries/regions separately, not by pooling what cannot be pooled.

**Response: Thanks. We largely agree with the reviewer. We have pointed out potential problems of pooling data when we discuss study limitations.** **Please see changes (lines 445-451) to the revised manuscript.**

**Part D: Reply to the comments from Reviewer #3**

Reviewer#3: Comments to the Author

**Comment 1**. I really enjoyed reading 'Impact of population ageing on global deaths, 1990-2017' manuscript.

**Response: Thanks for your time.**

**Comment 2**. The article aimed to provide a novel robust method to evaluate the global impact of population ageing across a lengthy time period, which it has done successfully in my opinion.

**Response: Thanks.**

**Comment 4**. The method showed in the manuscript is simple and robust as the authors claimed. The supplementary method is easy follow and it is sufficient for other researchers to reproduce. It'd be nice if the authors could expand the supplementary method to a tutorial with an actual example.

**Response: Thanks for the good suggestion. We have used the data from China as an example. Please see changes to the revised S1 Text.**

**Comment 5**. The manuscript is well organised and written clearly enough to be accessible to non-specialists. However, as a non native speakers I did not check any grammars.

**Response: Thanks. One of our co-authors is a native English speaker and has reviewed and edited the manuscript several times.**

**Comment 6**. More explanation of the method would help make the manuscript more complete without readers have to go to the supplementary. Word limits could be an issue but I think authors could shorten the discussion without losing anything important.

**Response: Thanks. As suggested, we have included the formulas for calculating main effects and interaction effects in the main text.** **Please see changes (lines 170-176) to the revised manuscript.**

**Part E: Reply to the comments from Reviewer #4**

Reviewer #4: Comments to the Author

**Comment 1**. Population ageing is obviously associated with increased rates of mortality. In this paper, the authors use data from the Global Burden of Disease to estimate the magnitude of this over the period 1990-2017. To do this, the authors use a novel statistical approach they recently developed that can decompose death differences between populations based on population size, age structure of the population, and age-specific mortality rate. The authors found wide differences across income levels, countries and causes of deaths.

**Response: Thanks for your time.**

**Comment 2**. Overall, I thought the paper was interesting and addressed an important topic. I have a few comments that I hope might improve the paper:

**Response: Thanks for the positive evaluation.**

**Comment 3**. 1. The authors could potentially report estimates for the increase in yearly deaths attributable to each year increase in a population's average age (controlling for income, country and other factors). Similar estimates could be given for increases in average income and changes in sex ratios. Such figures may be of interest to researchers in ageing and population planning.

**Response: Many thanks. We agree that more detailed analyses as suggested by the reviewer would be of great interest and value. However, we are unaware of an appropriate statistical method to support such analyses. The decomposition method used in this research attributes the change of total deaths to three factors – population growth, population ageing (change of age structure), and change of age-specific mortality. Theoretically, in our method, we could replace age groups by other more refined groups, e.g., age-sex groups or sex-income groups, and replace age-specific mortality rates by the refined group-specific mortality rates. However, such analyses would require much more refined data on population structures and mortality rates, which are not available. We will consider this possibility for future work, but this manuscript focuses on the traditional decomposition factors: population size, age structure and age-specific mortality rates. We also include it as a study limitation and a further research direction in the discussion.** **Please see changes (lines 445-451) to the revised manuscript.**

**Comment 4**. 2. The authors currently give breakdowns of mortality attributed to individual diseases by sex. It could potentially be helpful to researchers of specific diseases to also have estimates of a disease's contribution to mortality associated with population ageing independent of sex. While this could be difficult given differences in sex ratios across countries and, as the authors point out, with ageing, I wonder if the authors could use their decomposition approach to provide this.

**Response: Many thanks. We agree with the reviewer that sex may be an independent factor of mortality, and it would be valuable to explore the effect of sex and of changes in sex ratio in particular countries or for particular diseases. However, for the same reasons as stated in our response to the last comment, the decomposition method does not readily support analyses with both age and other factors such as sex or sex ratio with the currently available data. In fact, to partially control for the effect of sex, we present the results separated for each sex in the manuscript. In the future, we will think about how we may separate the effects of multiple factors that define population structures, given existing data.**

**Comment 5**. 3. In the section "Population Ageing", it could be helpful if the authors report the magnitude of ageing according to the income groupings (high, upper middle, lower middle, and low) that the authors use elsewhere in the results section.

**Response: Thanks for the good suggestion. We have added a table to show the information.** **Please see changes (lines 243-244) to the revised manuscript.**

**Comment 6**. 4. The authors report negative values in low income countries for the proportion of death increases attributable to population ageing. They explain: "The negative percentages in low-income countries reflect decreases in the proportion of old age groups" (p. 10). I found this slightly confusing - does this suggest that low-income countries' populations were negatively ageing (getting younger)? This point could be clarified in more detail.

**Response: Thanks. Yes, the reviewer is correct. Because the increase of younger people in these countries exceeded the increase of those aged 65 years and older between 1990 and 2017, the proportion of old people actually decreased from 3.2% in 1990 to 3.1% in 2017 (Table 1). Therefore, these low-income countries indeed become younger. We have improved the wording to make this point clear.** **Please see changes (lines 273-275, and lines 303-306) to the revised manuscript.**

**Comment 7**. 5. Given the novelty of their statistical method, I wonder if sensitivity analyses could provide converging evidence for their findings (e.g., giving very brief examples of countries that differ in age but with similar incomes and reporting the differences in mortality etc.).

**Response: Thanks for the good suggestion. In the paper we published previously (Cheng et al., 2019, PLoS One), we used several examples to demonstrate the robustness of the decomposition method compared to previously published methods. The validity of our method can also be seen by comparing Japan to the U.S., the former with a much faster ageing (proportion of 60+ increased from 11.9% in 1990 to 27.5 in 2017) than the latter (from 12.4% to 15.4%). The two countries are at the same income level. From Fig 3, we can see population ageing has a much higher attributed proportion in Japan than in the U.S., for both males and females, suggesting the reasonability of our method.**

**Comment 8**. 6. In the discussion, the authors discuss the tension between population ageing and mortality reduction from specific disease through prevention strategies and improved healthcare. This seems somewhat of a paradox to me - if mortality from a disease is reduced, I would have thought that would lead to further ageing, which, in turn, could eventually lead to mortality from other causes. Perhaps I am missing something, but maybe the authors could clarify this point and discuss this issue.

**Response: Many thanks for the good thought. The reviewer is correct; there is a bit of a paradox at play here. As we know, ageing has an impact on risk of death; death risk increases as an individual gets older. Not all diseases and injuries among older individuals can be easily prevented. As we prevent some types of diseases and injuries, others may emerge as more prominent while the population further ages. While prevention strategies and improved healthcare may not completely overcome the increased deaths from population ageing in all countries/territories, they certainly lead to a longer and more productive life. We have rephrased some related sentences in the discussion. Please see changes (lines 452-454, and lines 461-464) to the revised manuscript.**

**Comment 9**. The validity of the findings rests on a statistical approach that the authors themselves developed and published recently (Cheng et al., 2019, PLoS One). I do not have expertise in these statistics to evaluate this part of the paper. I would suggest that the editors obtain a review from a statistician in the area if they have not already.

**Response: Thanks. We cited the original work published by our team and included detailed formulas of the approach in the main text briefly and in the appendix at detail. In addition, a coauthor of the referred paper and this paper is a biostatistician from the University of Florida. We would be happy to answer any statistical questions concerning our approach.**
