# Supplementary material for: Population ageing and mortality during 1990–2017: A global decomposition analysis
Source: PLoS Med. 2020 Jun 8;17(6):e1003138. doi: 10.1371/journal.pmed.1003138 (PMC7279585; doi:10.1371/journal.pmed.1003138)
Supplement: Attachment — Submitted filename: Response to reviewers.docx [file pmed.1003138.s007.docx]

May 6, 2020

Dear Dr. McBride,

Many thanks for the final comments from you and the three reviewers. We greatly appreciate your time and are pleased to see a positive response to our work. Based on the requests and comments, we have worked to improve the manuscript. Meanwhile, we have carefully read the manuscript and have corrected the errors in Table 1 and for reference 20. In addition, we have corrected the format of manuscript based on the 2nd-round revised manuscript as the editor requests. We believe the manuscript is improved. Please find a point-by-point response to the editor’ requests and the reviewers’ comments below.

We hope the revised manuscript addresses the remaining concerns of the editor and reviewers.

Yours sincerely,

Correspondence to:

Prof. Guoqing Hu

Department of Epidemiology and Health Statistics

Xiangya School of Public Health, Central South University.

110 Xiangya Road, Changsha, 410078, China

Email: [huguoqing009@gmail.com](mailto:huguoqing009@gmail.com)

On behalf of all authors

**Part A: Reply to comments from the editor**

Requests from Editors:

**Comment 1**. 1- Abstract Background: “older people” or “older adults” rather than “old people”.

**Response: Thanks. we have changed it as suggested.**

**Comment 2**. 2- Abstract Methods and Findings and going forward, please consider using “high- and middle-income countries” and “low-income countries” rather than “countries with middle to high income levels”, unless these categories in your study do not line up with the WHO categories. If the latter is true, please make the distinction clear.

**Response: Thanks. Countries were classified according to the World Bank economic development level in this study. As suggested, we have altered our wording related to this term. Please see changes in the revised manuscript (line 36 and lines 42-43).**

**Comment 3**. 3- Abstract Methods and Findings, line 37: perhaps clearer to say “Compared to 1990, 12 million additional global deaths in 2017 were attributed to population ageing, corresponding to 27.9% of total global deaths.” (If my rewording is accurate).

**Response: Thanks. We have changed it as suggested.**

**Comment 4**. 4- Line 48: rather than “about 50%”, please state the absolute numbers for the numerator and denominator.

**Response: Thanks. As suggested, we have added the exact number. Please see changes in the revised manuscript (lines 54-55).**

**Comment 5**. 5- Starting in the Abstract and throughout the manuscript, please avoid causal language (e.g., “Population ageing caused increases in the number of deaths in countries with middle to high income levels but not for countries with low income”) and use “attributable for” or similar.

**Response: Thanks for the great suggestion. We have corrected the English writing as suggested. Please see changes to the revised manuscript.**

**Comment 6**. 6- Thank you for adding an Author Summary. Point number 7 (“The reduction in number of deaths from 1990 to 2017 from mortality change exceeds the increase of deaths caused by population ageing for the whole world and in about 50% of countries where population ageing was associated with increased death burden.”) is a bit confusing. It would be appropriate to split into two sentences to clarify.

**Response: Thanks. We have decided to keep this as a single sentence but have revised it to improve clarity. Please see changes to the revised manuscript (lines 93-98).**

**Comment 7**. 7- Please qualify the first sentence of the last paragraph of the Introduction with “to our knowledge” or similar.

**Response: Thanks. As suggested, we have changed it.**

**Comment 8**. 8- Line 167: please delete “robust”.

**Response: Thanks. As suggested, we have deleted it.**

**Comment 9**. 9- Line 189: please delete “new”.

**Response: Thanks. As suggested, we have deleted it.**

**Comment 10**. 10- Results section: thank you for including the absolute numbers that correspond to percentages in the tables. Please also include absolute numbers when citing the percentages in the text.

**Response: Thanks. As suggested, we have included absolute numbers in the main text.**

**Comment 11**. 11- Line 386: if you describe the decomposition method as “robust”, please explain here what makes it robust.

**Response: Thanks. As suggested in Comment 8, we have deleted it.**

**Comment 12**. 12- Again on lines 400, 407, and 426, please either describe how this method is robust or delete “robust”.

**Response: Thanks. As suggested, we have deleted it and provided more details in the revised manuscript (lines 82, 142, 147, 179, 411, 427, 434-435 and 455-456).**

**Part B: Reply to the comments from Reviewer #1**

Reviewer#1: Comments to the Author

**Comment 1**. The authors have addressed my concerns and I now recommend publication

**Response: Thanks.**

**Part C: Reply to the comments from Reviewer #2**

Reviewer#2: Comments to the Author

**Comment 1**. The authors have taken the comments seriously which resulted in a more cautious interpretation of their efforts.

**Response: Thanks.**

**Part D: Reply to the comments from Reviewer #4**

Reviewer#4: Comments to the Author

**Comment 1**. The authors have addressed my comments.

**Response: Thanks.**
